# Hydrogen sulfide and polysulfides induce GABA/glutamate/d-serine release, facilitate hippocampal LTP, and regulate behavioral hyperactivity

**DOI:** 10.1038/s41598-023-44877-y

**Published:** 2023-10-31

**Authors:** Hiroki Furuie, Yuka Kimura, Tatsuhiro Akaishi, Misa Yamada, Yoshiki Miyasaka, Akiyoshi Saitoh, Norihiro Shibuya, Akiko Watanabe, Naoki Kusunose, Tomoji Mashimo, Takeo Yoshikawa, Mitsuhiko Yamada, Kazuho Abe, Hideo Kimura

**Affiliations:** 1 Department of Neuropsychopharmacology, National Institute of Mental Health, National Center of Neurology and Psychiatry, Kodaira, Tokyo Japan; 2 Department of Pharmacology, Sanyo-Onoda City University, Sanyo-Onoda, Yamaguchi Japan; 3 Laboratory of Pharmacology, Faculty of Pharmacy and Research Institute of Pharmaceutical Sciences, Musashino University, Nishi-Tokyo, Tokyo Japan; 4 Departement of Medicine, Institute of Experimental Animal Sciences, Osaka University, Suita, Osaka Japan; 5 Department of Pharmacology, Faculty of Pharmaceutical Sciences, Tokyo University of Science, Noda, Chiba Japan; 6 Laboratory of Molecular Psychiatry, RIKEN Center for Brain Science, Wako, Saitama Japan; 7 School of Pharmaceutical Sciences, Kyushu University of Health and Welfare, Nobeoka, Miyazaki Japan; 8 Division of Animal Genetics, Laboratiry Animal Research Center, Institute of Medical Science, The Universtiry of Tokyo, Tokyo, Japan; 9 Department of Pathophysiology, Faculty of Human Nutrition, Tokyo Kasei Gakuin University, Chiyoda-ku, Tokyo, Japan

**Keywords:** Bioinorganic chemistry, Learning and memory, Long-term potentiation, Biochemistry, Cell biology, Chemical biology, Neuroscience, Neurology

## Abstract

Hydrogen sulfide (H_2_S) and polysulfides (H_2_S_n_, n ≥ 2) are signaling molecules produced by 3-mercaptopyruvate sulfurtransferase (3MST) that play various physiological roles, including the induction of hippocampal long-term potentiation (LTP), a synaptic model of memory formation, by enhancing N-methyl-d-aspartate (NMDA) receptor activity. However, the presynaptic action of H_2_S/H_2_S_n_ on neurotransmitter release, regulation of LTP induction, and animal behavior are poorly understood. Here, we showed that H_2_S/H_2_S_2_ applied to the rat hippocampus by in vivo microdialysis induces the release of GABA, glutamate, and d-serine, a co-agonist of NMDA receptors. Animals with genetically knocked-out 3MST and the target of H_2_S_2_, transient receptor potential ankyrin 1 (TRPA1) channels, revealed that H_2_S/H_2_S_2_, 3MST, and TRPA1 activation play a critical role in LTP induction, and the lack of 3MST causes behavioral hypersensitivity to NMDA receptor antagonism, as in schizophrenia. H_2_S/H_2_S_n_, 3MST, and TRPA1 channels have therapeutic potential for psychiatric diseases and cognitive deficits.

## Introduction

Hydrogen sulfide (H_2_S) and polysulfides (H_2_S_n_: n ≥ 2) have various physiological roles, including modulation of neurotransmission, vascular tone regulation, cytoprotection against oxidative stress, anti-inflammation, oxygen sensing, transcriptional regulation, and energy formation^1–3^. H_2_S is produced by cystathionine β-synthase (CBS), cystathionine γ-lyase (CSE), and 3-mercaptopyruvate sulfurtransferase (3MST)^4–9^. In the brain, 3MST is the primary enzyme to produce H_2_S^7^.

3MST also produces H_2_S_n_ and other S-sulfurated molecules (sulfane sulfur), such as cysteine persulfide, glutathione persulfide, and S-sulfurated cysteine residues, with 3-mercaptopyruvate (3MP) as the substrate^10–12^. 3MP is produced from l-cysteine and α-ketoglutarate by cysteine aminotransferase (CAT) and d-cysteine by d-amino acid oxidase^7,8^. A relatively high concentration of endogenous d-cysteine was recently measured in the embryonic brains^13^. 3MST is localized in both neurons and glia^7,8,14^, and the total levels of sulfane sulfur in the brains of 3MST-KO mice are less than half of those in wild-type rats, suggesting that 3MST is a significant enzyme that produces sulfane sulfur in the brain^11^.

S-sulfuration (S-sulfhydration), which adds sulfur (S) to the thiols of cysteine residues, has been proposed as a mode of action of H_2_S to regulate the activity of target proteins^15^. Under physiological conditions, approximately 10% of cysteine residues of proteins are oxidized^16^ such as in the forms of cysteine disulfide bond (R-Cys-S-S-Cys-R), S-nitrosylated—(R-Cys-SNO), and S-sulfinated—(R-Cys-SOH) residues, of which sulfur has an oxidation state of − 1 or 0^1,2^. Because sulfur atoms in the same oxidation state do not react with each other, these oxidized cysteine residues are S-sulfurated (R-Cys-SSH) by H_2_S (oxidation state: − 2). In contrast, sulfur in reduced cysteine residues (R-Cys-SH) has an oxidation state of − 2 and is sulfurated by H_2_S_2_ and other polysulfides (oxidation state: − 1 or 0)^1,2^. For example, the first step in the metabolism of H_2_S in mitochondria is reducing the cysteine disulfide bond (R-Cys-S-S-Cys-R) of sulfide quinone oxidoreductase (SQR)^17^. At the same time, H_2_S_2_ and H_2_S_3_ activate transient receptor potential ankyrin 1 (TRPA1) channels by sulfurating two cysteine residues localized to the amino terminus of the channels^18–20^. The activation of TRPA1 channels was initially reported as an effect of H_2_S, but it was later found that the potency of H_2_S_2_ and H_2_S_3_ far exceeded that of H_2_S^21–23^.

H_2_S facilitates the induction of hippocampal long-term potentiation (LTP) by enhancing the activity of N-methyl-d-aspartate (NMDA) and reducing the cysteine disulfide bond localized to the ligand binding domain^5,24^. Despite having weaker reducing activity than dithiothreitol (DTT), H_2_S facilitates LTP induction more than DTT^5^. This observation suggests an additional mechanism for H_2_S to facilitate LTP induction. We found that H_2_S_n_ activate TRPA1 channels by sulfurating two cysteine residues localized at the amino terminus to induce Ca^2+^ influx in astrocytes, which release gliotransmitters to modulate synaptic activity^18–23^. Shigetomi et al. reported that the activation of TRPA1 channels in astrocytes is required to induce LTP and that its mechanism is the release of d-serine, a co-agonist of NMDA receptors^25^. However, the release of d-serine from astrocytes is controversial, and further studies on the involvement of TRPA1 channels in LTP induction, including the effect of H_2_S_n,_ are awaited^26^.

Only the toxic effects of high concentrations of H_2_S on decreasing the levels of neurotransmitters in the brain have been reported^27,28^. However, the effects of physiological concentrations of H_2_S and H_2_S_n_ on intracellular levels and neurotransmitter release are poorly understood.

The involvement of H_2_S in the pathogenesis and cognitive deficits of schizophrenia has been reported^29–31^. Deficiency in 3MST causes mercapto lactate-cysteine disulfiduria, which is associated with mental retardation^32^. 3MST-KO mice showed anxiety-like behaviors, a schizophrenia-related behavior^33^, while TRPA1 channels were involved in anxiety-like behaviors^34,35^.

Abnormalities in the balance between glutamatergic and GABAergic activities have been implicated in the pathophysiology of schizophrenia^36–38^. GABA_A_ receptors regulate extracellular d-serine concentrations, and serine racemase (SR), an enzyme that converts l-serine to d-serine, is associated with susceptibility to schizophrenia in humans and a mouse model^39,40^. The hyperlocomotion induced by NMDA antagonists in rodents is thought to reflect the capacity of drugs to provoke psychosis in humans^41^. Postnatal ablation of NMDA receptors on GABAergic interneurons enhances behavioral sensitivity to a non-competitive NMDA receptor antagonist, MK-801, as in schizophrenia^42^.

In this study, we discovered that H_2_S and H_2_S_2_ induces the release of GABA, glutamate, d-serine, and other transmitters. Neurotransmitters regulate the release of H_2_S and polysulfides. The effects of H_2_S and H_2_S_2_ produced by 3MST and the activation of TRPA1 channels facilitate the induction of LTP. Lacking in 3MST augmented hyperlocomotion induced by MK-801.

## Results

### Establishment of 3MST-knockout (KO), TRPA1-KO, and 3MST/TRPA1 double KO rats and their tissue morphology

**Figure 1:**
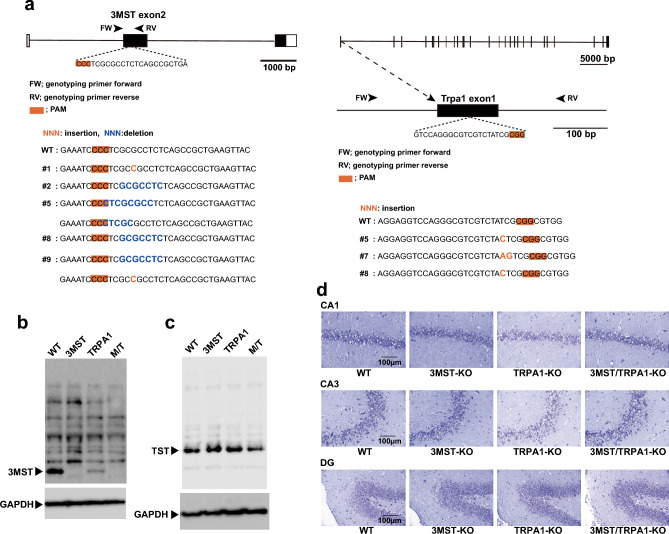
Generation of 3MST-KO, TRPA1-KO, and 3MST/TRPA1-double-KO rats, their morphology, the levels of 3MST and TST. **a**. Schematic representation of rat 3MST and TRPA1 gene structures and sequences of wild-type and mutant alleles. Mutants 3MST #5 and TRPA1 #8 were established and used in the present study. **b** and **c**. Western blot analysis of the brains of wild-type, 3MST-KO, TRPA1-KO, and 3MST/TRPA1 double-KO rats, using antibodies against 3MST (**b**) and rhodanese or thiosulfate sulfurtransferase (TST) (**c**). (**b**) and (**c**) are images cropped from the original blots presented in Supplementary Fig. 2. **d**. Cresyl violet staining of the hippocampal slices. The CA1, CA3, and dentate gyrus (DG) obtained from 3MST-KO, TRPA1-KO, 3MST/TRPA1-double-KO, and wild-type rats were stained.

3MST-KO and TRPA1-KO rats were developed to examine the effect of 3MST and TRPA1 channels on the release of neurotransmitters, the induction of LTP, and the behavior. 3MST/TRPA1-double-KO rats were obtained by mating 3MST-KO with TRPA1-KO rats until both alleles of 3MST and TRPA1 were mutated (Supplementary Fig. 1). Using CRSPR-Cas9 technology, we inserted sequences into or deleted them from exon 2 of the 3MST gene for 3MST-KO production, while only inserting sequences into exon 1 of the TRPA1 gene to generate TRPA1-KO (Fig. 1a). These insertions or deletions caused a frameshift in the coding regions of the 3MST and TRPA1 genes. We mated 3MST-KO rats with the #5 construct and TRPA1-KO rats with #8 for the present study until both alleles of each gene were mutated (Fig. 1a; Supplementary Fig. 1).

Western blot analysis with an antibody against 3MST showed that the brains of wild-type rats and TRPA1-KO had a band corresponding to 3MST, while those of 3MST-KO and 3MST/TRPA1 double-KO rats did not (Fig. 1b, Supplementary Fig. 2a–h). The level of 3MST in TRPA1-KO rats was lower than that in wild-type rats. The rhodanese or thiosulfate sulfurtransferase (TST) levels increased in previously reported 3MST-KO mice, in which the transcriptional regulation regions of both genes overlapped^43^. In contrast, in the present study, TST levels did not change in 3MST-KO rats whose control regions were separated (Fig. 1c, supplementary Fig. 2i–p).

It is challenging to detect TRPA1 channels by western blot analysis because of the lack of an antibody specific and sensitive enough to detect channels in the brain, as reported previously^44^.

Morphological differences were examined in the CA1, CA3, and dentate gyrus (DG) of the hippocampus, cerebellum, cerebral cortex, lung, and kidney. No specific morphological differences were observed between tissues (Fig. 1d and Supplementary Fig. 3).

### The endogenous levels of H_2_S, H_2_S_n_, cysteine, and glutathione in the hippocampus

**Figure 2:**
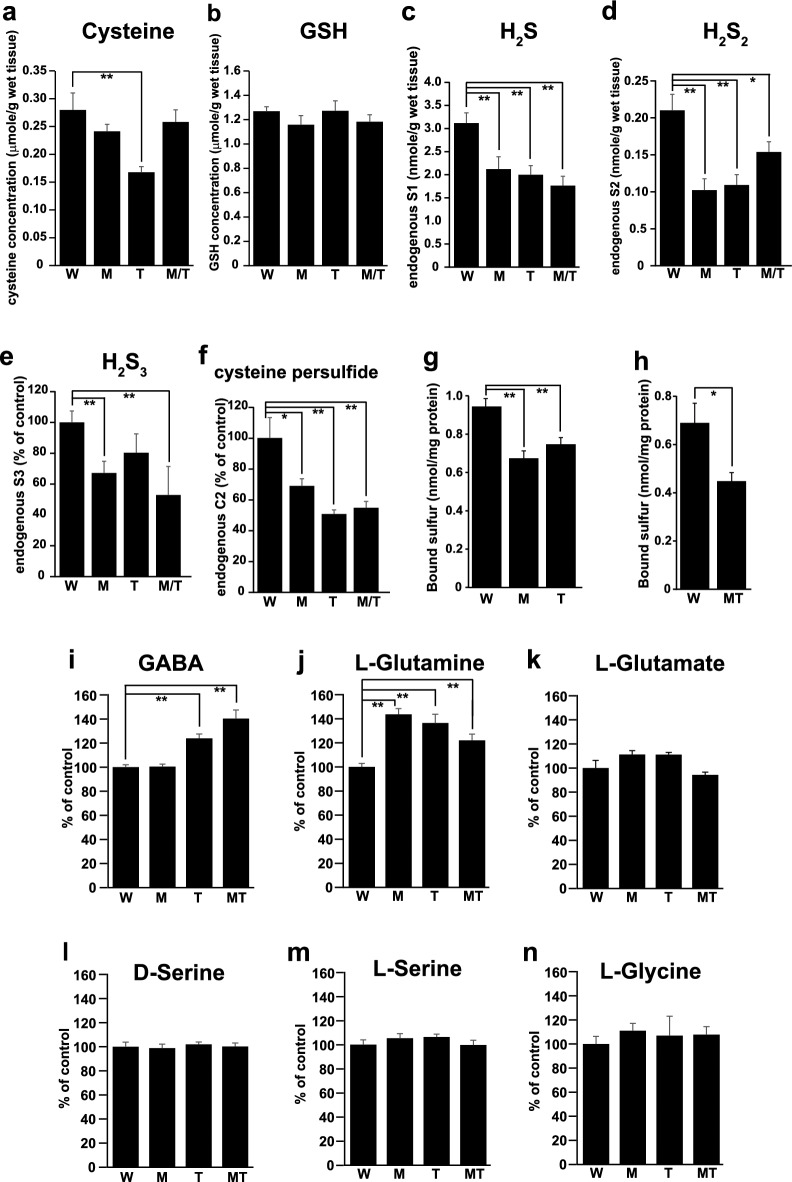
The intracellular levels of cysteine, glutathione, H_2_S, H_2_S_2_, H_2_S_3_, cysteine persulfide, bound sulfane sulfur, and transmitters in KO rats compared to those of the wild-type. **a** and **b**. The intracellular concentrations of cysteine (**a**) and glutathione (**b**) in the hippocampus measured by HPLC. **c** to **f**. Intracellular levels of H_2_S (**c**), H_2_S_2_ (**d**), H_2_S_3_ (**e**), and cysteine persulfide (**f**) in the hippocampus were measured using LC–MS/MS. **g** and **h**. Intracellular levels of bound (sulphane) sulfur in the hippocampus were measured by gas chromatography. **i** to **n**. Relative intracellular concentrations of GABA (**i**), l-glutamine (**j**), l-glutamate (**k**), d-serine (**l**), l-serine (**m**), and l-glycine (**n**) in the hippocampus measured by HPLC. W, wild-type; M, 3MST-KO; T, TRPA1-KO; MT, 3MST/TRPA1-double-KO. The experiments were repeated five times. *p < 0.05, **P < 0.01 significantly different as indicated by the Student's t-test. All data are shown mean ± SEM.

Endogenous levels of cysteine and glutathione in the hippocampus were measured using HPLC, and those of H_2_S, H_2_S_2_, H_2_S_3_, and cysteine persulfide were detected using LC–MS/MS (Fig. 2a–f, Supplementary Fig. 4a,b). The reaction of monobromobimane (MBB) with thiols was performed under alkaline conditions (pH 9.5), which increased levels of H_2_S while decreasing polysulfides^45^. In the present study, an improved method was used in which the reaction of MBB with thiols was performed at physiological pH 7.0^12^. This method enabled us to confirm cysteine and glutathione levels using HPLC.

The levels of cysteine in TRPA1-KO were approximately 60.0% of those in the wild-type, whereas those in 3MST-KO and 3MST/TRPA1-double-KO were not significantly different from those in the wild-type (Fig. 2a). A similar result has been reported in a defective mutant of TRPA1 channels in Drosophila, in which the level of cysteine is 0.50-fold that in the wild-type^46^. The amount of glutathione was not significantly different between KOs and the wild type (Fig. 2b).

The levels of H_2_S were lower in all three KO hippocampi than in the wild type (Fig. 2c), and those of H_2_S_2_ were the lowest in 3MST-KO rats, approximately half of those in the wild type (Fig. 2d). TRPA1-KO and 3MST/TRPA1-double-KO rats also showed lower levels of H_2_S_2_. The 3MST-KO and 3MST/TRPA1-double KO rats showed lower levels of H_2_S_3_ than wild-type rats, and all three KO rats had lower levels of cysteine persulfide (Fig. 2e,f).

### Bound (sulfane) sulfur levels in the brain

Bound (sulfane) sulfur, which consists of S-sulfurated molecules such as H_2_S_n_, cysteine persulfide, and S-sulfurated cysteine residues, releases the corresponding amount of H_2_S under reducing conditions^7,8,47–49^. The levels of bound sulfur in the brains of 3MST-KO, TRPA1-KO, and 3MST/TRPA1-double KO rats were lower than those in the brains of wild-type rats (Fig. 2g,h, Supplementary Fig. 4c). We previously obtained similar results in 3MST-KO mice^11^. The levels of bound sulfur reflected the lower levels of H_2_S_2_, H_2_S_3_, and cysteine persulfide in KO rats, as measured by LC–MS/MS (Fig. 2d–f).

### Endogenous levels of neuro- and glio-transmitters

The endogenous levels of GABA, glutamine, glutamate, d-serine, l-serine, and glycine in the hippocampus were measured by HPLC. GABA levels were significantly higher in TRPA1-KO and 3MST/TRPA1-double-KO rats than in wild-type and 3MST-KO rats (Fig. 2i). This result agrees well with the observation that GABA levels are 1.44 times greater in TRPA1 defective mutant of Drosophila mutants than in the wild-type^46^. The levels of glutamine, which is an essential precursor for neurotransmitters glutamate and GABA, were greater in all three types of KOs than those in the wild-type, while those of glutamate were slightly higher in 3MST-KO and TRPA1-KO but not statistically significant (Fig. 2j,k).

Endogenous d-serine, l-serine, and glycine levels were not significantly different between the KO and wild-type rats (Fig. 2l–n).

### H_2_S and H_2_S_n_ induced the release of transmitters from suspended brain cells

To examine whether H_2_S, H_2_S_2_, and H_2_S_3_ induce the release of neurotransmitters and related amino acids from brain cells, 20 μM each of Na_2_S, Na_2_S_2_, and Na_2_S_3_, sodium salts of H_2_S, H_2_S_2_, and H_2_S_3_, respectively, were applied to the brain cell suspension prepared from 7 to 14 day-postnatal KOs and wild-type rats.

**Figure 3:**
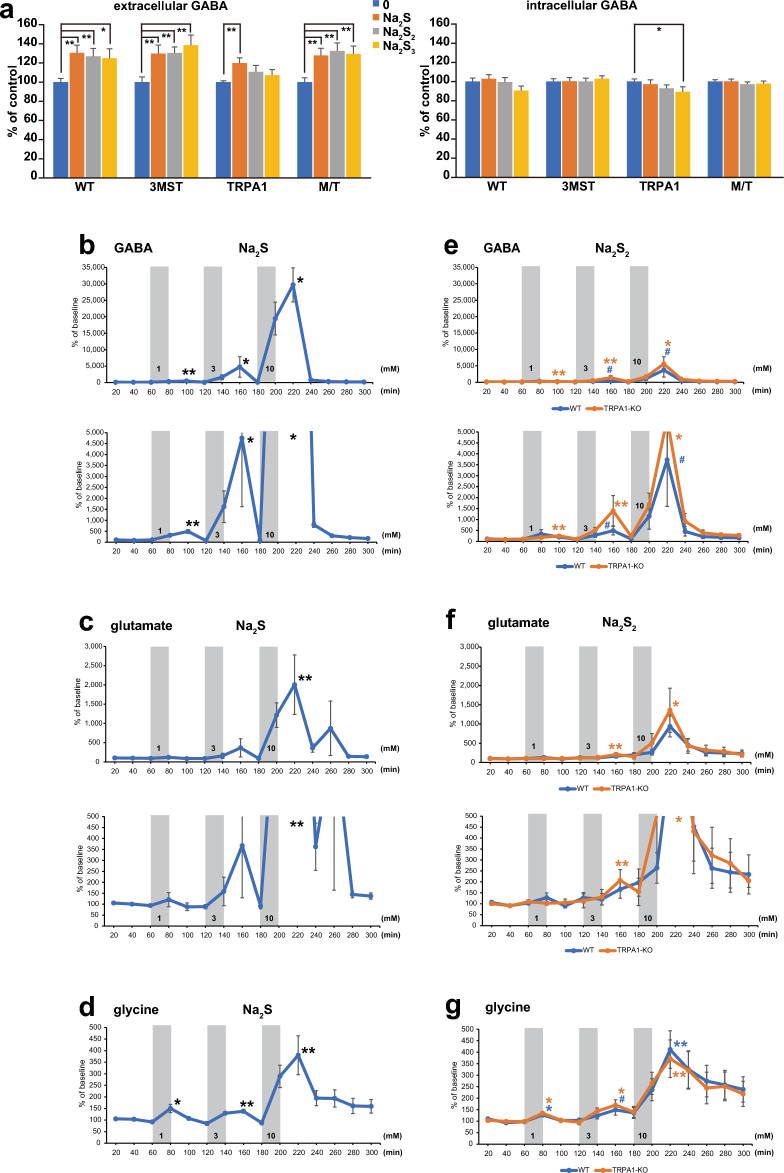
The release of GABA, glutamate and glycine induced by H_2_S and H_2_S_n_ from the brain cell suspension and in in vivo microdialysis. **a**. The release of GABA from the brain cell suspension stimulated by 20 μM each of Na_2_S, Na_2_S_2,_ and Na_2_S_3_ and the changes in the intracellular GABA levels. * p < 0.05, **p < 0.01 with Student's t-test. **b** to **g**. The release of GABA (**b** and **e**), glutamate (**c** and **f**), glycine (**d** and **g**) induced by H_2_S (**b** to **d**) and by H_2_S_2_ (**e** to** g**) at the concentrations indicated in the figure in the wild-type rats (blue line) and TRPA1-KO (orange line). A baseline is defined as 100. (**b)**, n = 5; (**c**) and (**d**) n = 7; (**e**) the wild-type n = 5, TRPA1-KO n = 8; (**f**) the wild-type n = 6, TRPA1-KO n = 9; (**g**) the wild-type n = 9, TRPA1-KO n = 8; All data are shown means ± SEM. Each peak value of the response was compared to the corresponding base line response observed at the start of the application of Na_2_S or Na_2_S_2_. *p < 0.05, **p < 0.01, ^#^p < 0.1 with Student’s t-test.

GABA was released into the extracellular milieu by applying Na_2_S, Na_2_S_2,_ and Na_2_S_3_. However, the levels induced by Na_2_S_2_ and Na_2_S_3_ in TRPA1-KO rats were lower than those in the other KO and wild-type rats (Fig. 3a). In contrast, the intracellular GABA concentration did not change significantly (Fig. 3a). GABA is efficiently released into the extracellular milieu via H_2_S, H_2_S_2,_ and H_2_S_3_.

The release of glycine induced by H_2_S from cells prepared from TRPA1-KO rats and H_2_S_2_ and H_2_S_3_ from 3MST-KO rats was significantly increased (Supplementary Fig. 5a). The release of d-serine, l-serine, glutamine, or glutamate was not detected in the presence of H_2_S, H_2_S_2_ or H_2_S_3_ (Supplementary Fig. 5b–e).

The release of d-serine, l-serine, and glutamine by H_2_S, H_2_S_2,_ and H_2_S_3_ may require interactions between neurons and glia. However, the cells were dispersed in the cell suspension and may not have had sufficient interactions between the cells. Therefore, it is necessary to examine transmitters' release into adult rats' hippocampi in vivo.

### H_2_S and H_2_S_2_ stimulate the release of transmitters in in-vivo microdialysis of the hippocampus

We examined the release of neurotransmitters and gliotransmitters induced by H_2_S and H_2_S_2_ in *in-vivo* microdialysis in the hippocampi of adult rats. Since the in vitro recovery from microdialysis probes has been reported between 0.1 and 7.6% depending on molecules^50^, we examined it on Na_2_S and Na_2_S_2_. Na_2_S was recovered approximately 0.2–1% and Na_2_S_2_ was 0.8–4.8% (Supplementary Table 1). Based on these observations, a minimum of 50 times higher concentration (1 mM) of Na_2_S and Na_2_S_2_ than that used for the brain cell suspension (20 μM) was applied.

One mM Na_2_S released GABA, glutamate, and glycine at approximately 3.2-, 1.2, and 1.5 times, respectively, of the baseline, and each release increased in a concentration-dependent manner up to approximately 300, 20, and 4 times, respectively, at 10 mM in wild-type rats (Fig. 3b–d). After the cessation of the Na_2_S application, the release returned to the baseline. During the experiments, no specific behavioral changes were observed in any rat.

**Figure 4:**
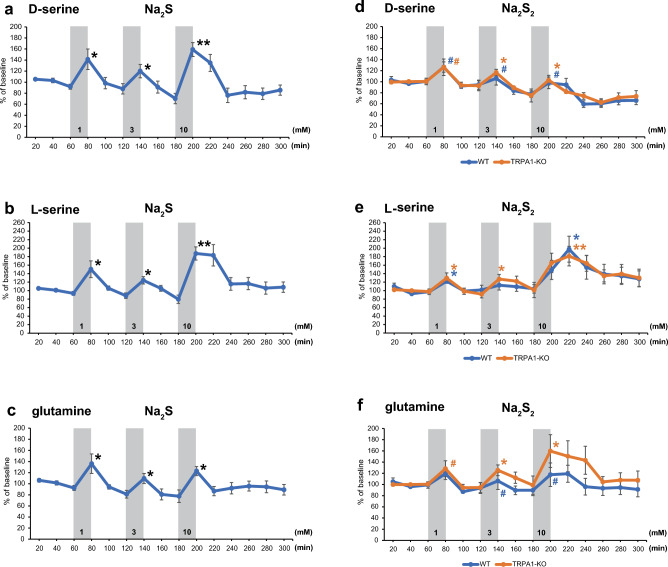
The release of d-serine, l-serine and glutamine induced by H_2_S and H_2_S_2_ in in vivo microdialysis. **a** to **f**. The release of d-serine (**a** and **d**), l-serine (**b** and **e**), glutamine (**c** and **f**) induced by H_2_S (**a** to **c**) and by H_2_S_2_ (**d** to** f**) at the concentrations indicated in the figure in the wild-type rats (blue line) and TRPA1-KO (orange line). A baseline is defined as 100. (**a**) to (**b**) n = 7; (**d**) to (**f**) the wild-type n = 9, TRPA1-KO n = 8. All data are shown means ± SEM. Each peak value of the response was compared to the corresponding base line response observed at the start of the application of Na_2_S or Na_2_S_2_. *p < 0.05, **p < 0.01, ^#^p < 0.1 with Student’s t-test.

Na_2_S_2_ also induced the release of GABA, glutamate, and glycine from both wild-type and TRPA1-KO rats but was less efficient than Na_2_S (Fig. 3b–g).

d-serine, l-serine, and glutamine were also released by 1 mM Na_2_S, which was approximately 1.4-, 1.5, and 1.4 times than the baseline, respectively, and 1.6-, 1.9, and 1.2 times, respectively, at 10 mM in wild-type rats (Fig. 4a–c). Similar results were obtained for the effect of Na_2_S_2_ with no significant difference between wild-type and TRPA1-KO rats (Fig. 4d–f).

These observations suggest that H_2_S and H_2_S_2_ induce the release of d-serine, l-serine, and glutamine less efficiently than that of GABA, glutamate, and glycine.

### The regulation of the release of H_2_S and polysulfides by neurotransmitters

**Figure 5:**
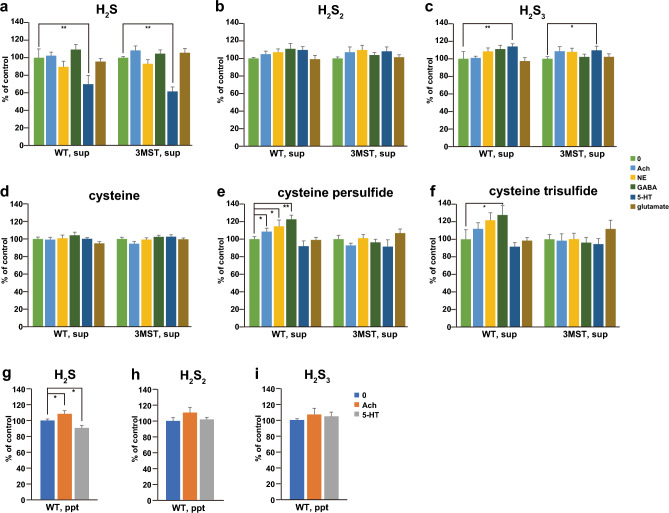
Serotonin and GABA regulate a release of H_2_S, H_2_S_2_, H_2_S_3,_ and cysteine persulfide. **a** to **f**. Suspended brain cells prepared from the wild-type and 3MST-KO rats were stimulated by 200 μM each of acetylcholine, norepinephrine, GABA, Serotonin (5HT), and glutamate, and released H_2_S (**a**), H_2_S_2_ (**b**), H_2_S_3_(**c**), cysteine (**d**), cysteine persulfide (**e**), and cysteine trisulfide (**f**) to the medium were measured. **g** to **i**. Suspended brain cells prepared from the wild-type rats were stimulated by 200 μM each of acetylcholine and 5HT, and the intracellular levels of H_2_S (**g**), H_2_S_2_ (**h**), and H_2_S_3_ (**i**) were measured. The experiments were repeated 8–12 times. * p < 0.05, ** p < 0.01 by student t-test. All data are shown mean ± SEM.

We also examined whether neurotransmitters induced the release of H_2_S, H_2_S_2_, H_2_S_3_, and cysteine persulfide in the brain cell suspensions. Serotonin significantly decreased the release of H_2_S to approximately 70% of the control in both the wild-type and 3MST-KO brain cell suspensions (Fig. 5a). Serotonin also decreased the intracellular levels of H_2_S to approximately 90% of the control in the wild-type (Fig. 5g). In comparison, it significantly increased the release of H_2_S_3_ to approximately 114% of that control (Fig. 5c).

GABA significantly increased the release of cysteine persulfide and cysteine trisulfide from the wild-type but did not induce their release from those prepared from 3MST-KO rats (Fig. 5e and f). GABA also increased the release of H_2_S, H_2_S_2_, and H_2_S_3,_ but the increase was not significant (Fig. 5a–c). As the molecular weight of cysteine persulfide was too close to that of dopamine to be differentiated with the LC–MS/MS used in the present study, the effect of dopamine was not examined.

Acetylcholine significantly increased the release of cysteine persulfide and the intracellular concentration of H_2_S in the wild type (Fig. 5e and g). Norepinephrine significantly increased the release of cysteine persulfide in wild-type rats but not in 3MST-KO rats (Fig. 5e).

These results were also examined using in vivo microdialysis. However, the release of H_2_S, H_2_S_2_, H_2_S_3_, or cysteine persulfide was not detected, probably because of the interaction of the dialysis membrane with low levels of released H_2_S and H_2_S_n_ (Supplementary Table 1).

### Comparison of responses of neurons to NMDA

**Figure 6:**
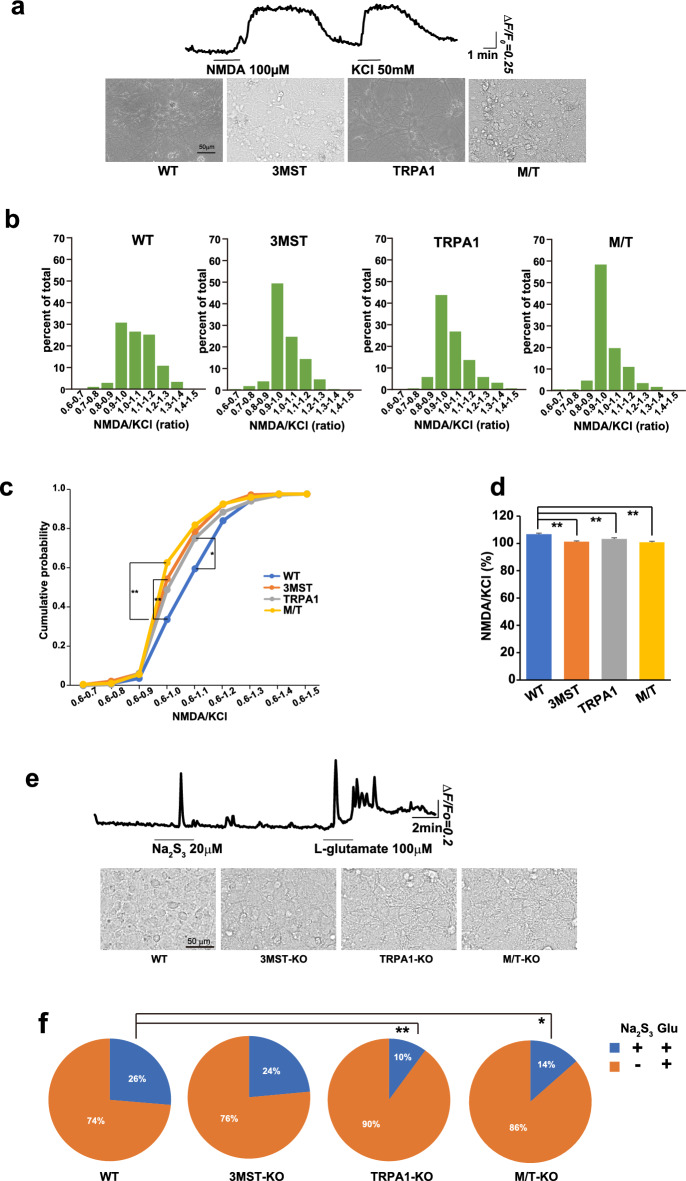
Sensitivity to transmitters of neurons and astrocytes prepared from 3MST-KO, TRPA1-KO, and 3MST/TRPA1-double-KO compared to those from the wild-type rats. **a** and **b**. Responses of neurons to N-methyl-d-aspartate (NMDA) and high K^+^ measured by Ca^2+^ imaging. (**a**) Typical response to 100 μM NMDA and 50 mM KCl (upper panel) and images of cultured neurons prepared from KO and wild-type rats (lower panel). (**b**) The ratio of responses to NMDA/ high K^+^ stratified by amplitude (F/F_0_). **c**. Kolmogorov–Smirnov cumulative analysis of NMDA/KCl. **p < 0.01, *p < 0.05. **d**. Student's t-test was used to determine the NMDA/KCl ratio (%) of total cells. WT: 106 ± 0.70, 3MST-KO: 101 ± 0.67, TRPA1-KO: 103 ± 0.86, 3MST/TRPA1-double KO: 100 ± 0.75. **p < 0.01. (**e**) A typical response of astrocytes to 20 μM Na_2_S_3_ and 100 μM glutamate (upper panel) and images of cultured astrocytes prepared from KO and wild-type rats (lower panel). (**f**) percentage of astrocytes responding to Na_2_S_3_ and glutamate (blue), and glutamate alone (orange). The percentage of cells responded to Na_2_S_3_ and glutamate in TRPA1-KO and in M/T-KO is significantly different from that in WT cells by the Chi-squared-test, while that of TRPA1-KO is not significantly different from that of M/T-KO. **p < 0.01 and *p < 0.05. Note that cells responded to Na_2_S_3_ but not to glutamate were observed 9 out of 423 cells in the wild-type, 5 out of 583 in 3MST-KO, 2 out of 210 in TRPA1-KO, and 0 out of 222 in 3MST/TRPA1-double KO. The experiments were repeated at least ten times. The total cells measured were 173–331 (**a**–**d**) and 210–583 (**f**).

The sensitivity of neurons to NMDA and high K^+^ levels was examined in primary cultures of hippocampal neurons using Ca^2+^ imaging (Fig. 6a–d, Supplementary Fig. 6a,b). The percentage of neurons with strong responses (higher ΔF/F0 values of responses) to NMDA and 50 mM K^+^ was slightly greater in wild-type rats than in all three types of KO rats (Supplementary Fig. 6a,b). Two-sample Kolmogorov–Smirnov cumulative analysis of NMDA/KCl showed that 3MST-KO and 3MST/TRPA1-double-KO has the significantly (p < 0.01) larger number of cells with small ratios (0.6–1.0) compared to the wild-type, and a similar observation was obtained for TRPA1-KO at 0.6–1.1 (p < 0.05) (Fig. 6b,c). Student's t-test of the ratio for total cells showed that neurons prepared from the wild-type exhibited slightly but significantly (p < 0.01) greater responses to NMDA than those prepared from the three types of KO (Fig. 6d).

### The responses of astrocytes to Na_2_S_3_ and glutamate

We previously showed that H_2_S_2_ and H_2_S_3_ activate TRPA1 channels in astrocytes and dorsal root ganglion cells^18,20^. The sensitivity of astrocytes to H_2_S_3_ and glutamate was examined by Ca^2+^ imaging (Fig. 6e,f)^18,51^. Astrocytes prepared from the wild-type rats and 3MST-KO rats responded to Na_2_S_3_ and glutamate (Fig. 6f). In contrast, astrocytes prepared from TRPA1-KO and 3MST/TRPA1-double-KO responded to Na_2_S_3_ in a smaller percentage of cells than those prepared from wild-type and 3MST-KO rats (Fig. 6f). Responses to Na_2_S_3_ of astrocytes prepared from TRPA1-KO and 3MST/TRPA1-double KO rats may be mediated by other channels, including TRP Vanilloid 4, which also respond to H_2_S_n_^23^.

### The induction of LTP

Because H_2_S and H_2_S_2_ facilitate the release of transmitters (Fig. 3), and because the activation of TRPA1 channels^18,20^ by H_2_S_2_ and their involvement in the induction of LTP has been proposed^25^, the effect of exogenously applied H_2_S_2_ on LTP induction was investigated.

**Figure 7:**
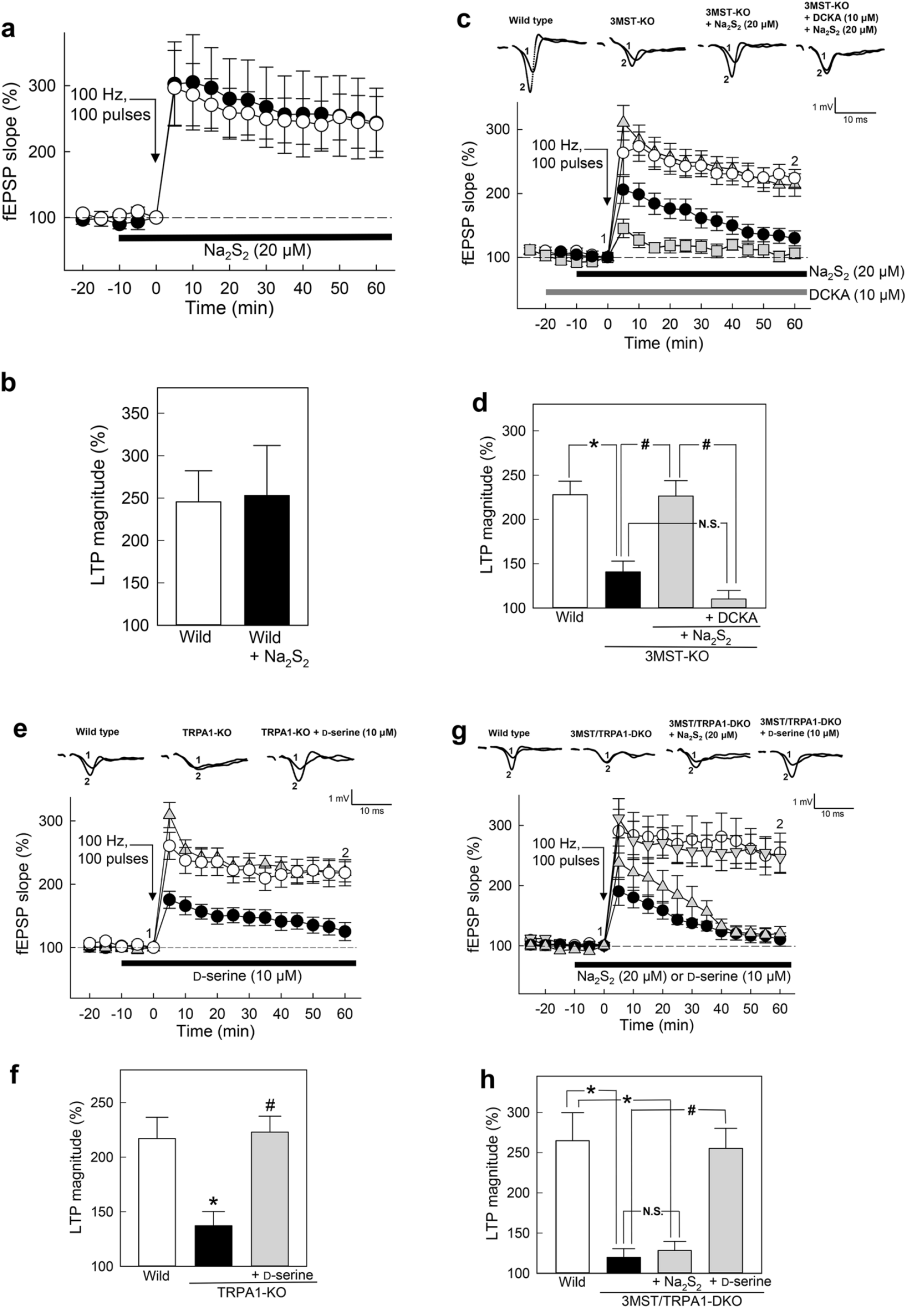
Induction of LTP in the hippocampus of 3MST-KO, TRPA1-KO, 3MST/TRPA1-double-KO and the wild-type rats. **a** and **b**. LTP induced by high-frequency stimulation (100 pulses at 100 Hz) in the wild-type in the presence (closed circles, n = 4) or absence (open circles, n = 5) of 20 μM Na_2_S_2_. Time course of changes in the fEPSP slope (**a**). The data in (**a**) were summarized by calculating the average of fEPSP slopes 30–60 min after high-frequency stimulation as an index of LTP magnitude (**b**). **c** and **d**. LTP induced in 3MST-KO in the presence or absence of 20 μM Na_2_S_2_ (Wild: open circles, n = 11; 3MST-KO: closed circles, n = 11; 3MST-KO + Na_2_S_2_: gray triangles, n = 11) and 10 μM 5,7-dichlorokynurenic acid (DCKA) (3MST-KO + Na_2_S_2_ + DCKA: gray squares, n = 11). Time course of changes in the fEPSP slope (**c**). The data in (**c**) was summarized as an index of LTP magnitude (**d**). One-way ANOVA followed by Tukey’s test (*F*_3_, _40_ = 18.232, P < 0.001). **e** and **f**. LTP induced in TRPA1-KO in the presence or absence of 10 μM d-serine (Wild: open circles, n = 10; TRPA1-KO: closed circles, n = 10; TRPA1-KO + d-serine: gray triangles, n = 10). Time course of changes in the fEPSP slope (**e**). The data in (**e**) was summarized as an index of LTP magnitude (**f**). One-way ANOVA followed by Tukey’s test (*F*_2_, _27_ = 8.997, P = 0.001). **g** and **h**. LTP induced in 3MST/TRPA1-double KO in the presence or absence of 20 μM Na_2_S_2_ (3MST/TRPA1-DKO: closed circles, n = 11; 3MST/TRPA1-DKO + Na_2_S_2_: gray triangles, n = 11) and 10 μM d-serine (Wild: open circles, n = 11; 3MST/TRPA1-DKO + d-serine: gray inverted triangles, n = 11). Time course of changes in the fEPSP slope (**g**). The data in (**g**) was summarized as an index of LTP magnitude (**h**). Kruskal–Wallis ANOVA on Ranks followed by Tukey’s test (*H* = 26.727, P < 0.001).

The effect of exogenously applied H_2_S_2_ on LTP induction in the CA1 area of hippocampal slices was investigated in wild-type rats. The magnitude of LTP induced by high-frequency stimulation was not significantly affected by applying Na_2_S_2_ (Fig. 7a,b). This observation suggests that endogenous H_2_S_2_ and high-frequency stimulation are sufficient to induce LTP in wild-type hippocampal slices.

#### LTP in 3MST-KO

To further examine the involvement of H_2_S_2_ in LTP induction, the LTP levels in 3MST-KO rats were compared with those in wild-type rats. While applying high-frequency stimulation generated robust LTP in wild-type rats, LTP was significantly reduced in 3MST-KO rats (Fig. 7c,d). However, it recovered to the level induced in the wild type in the presence of 20 μM Na_2_S_2_ (Fig. 7c,d), suggesting that H_2_S_2_ produced by 3MST is required to facilitate LTP induction.

Because H_2_S and H_2_S_2_ induce the release of d-serine, the effect of 5,7-dichlorokynurenic acid (DCKA), a specific inhibitor of the glycine (d-serine)-binding site in NMDA receptors, on the induction of LTP was examined. Even in the presence of 20 μM Na_2_S_2_, LTP was greatly suppressed by DCKA in 3MST-KO rats (Fig. 7c,d). This observation confirms that d-serine plays a role in the induction of LTP downstream of H_2_S_2_.

The involvement of endogenous H_2_S and H_2_S_2_ produced by 3MST in basal synaptic transmission and short-term plasticity was examined by comparing the input–output relationship and paired-pulse facilitation. There was no significant difference in the responses between 3MST-KO and wild-type rats (Supplementary Fig. 7a,b), indicating that basal synaptic transmission in 3MST-KO rats was normal and that the reduced LTP could not be due to differences in basal synaptic efficiency.

#### LTP in TRPA1-KO

Since H_2_S_2_ facilitates LTP induction, the involvement of its target protein, TRPA1 channels, in LTP induction was examined. The magnitude of LTP measured in TRPA1-KO rats was greatly reduced compared to that in wild-type rats (Fig. 7e). In contrast, the application of d-serine recovered the LTP to the level observed in the wild-type strain. These observations agree with previous findings^25^ that activating TRPA1 channels is critical for LTP induction (Fig. 7e,f, Supplementary Fig. 7c), although the release of d-serine is not dependent on TRPA1 channels (Fig. 4d).

The basal synaptic transmission in TRPA1-KO rats is normal and that the difference in basal synaptic efficiency cannot cause the reduced LTP (Supplementary Fig. 7d,e).

#### LTP in 3MST/TRPA1-double-KO

The effect of deficiency in both H_2_S_2_ production and TRPA1 channel activity on the induction of LTP was examined in 3MST/TRPA1-double-KO rats and compared with that in wild-type rats. The magnitude of LTP in 3MST/TRPA1-double-KO was greatly reduced (Fig. 7g). Application of 20 μM Na_2_S_2_ did not significantly recover LTP (Fig. 7g,h). In contrast, the application of 10 μM d-serine restored the magnitude of LTP to levels not significantly different from those of the wild-type (Fig. 7g,h), suggesting that d-serine application compensated for the lack of 3MST and TRPA1 channels.

The basal synaptic transmission in 3MST/TRPA1-double-KO was normal and that the difference in basal synaptic efficiency could not cause the reduced LTP (Supplementary Fig. 7f,g).

### Hyperlocomotion induced by MK-801 in 3MST-KO rats

**Figure 8:**
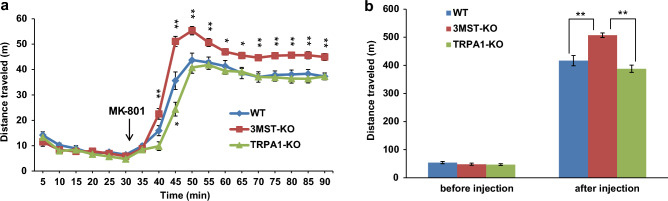
Hyperlocomotion induced by MK-801 in 3MST-KO rats. **a**. Time course of the distance traveled by the wild-type (WT) (n = 12), 3MST-KO (n = 12), and TRPA1-KO (n = 12) rats before and after administration of MK-801(0.2 mg/kg). Two-way ANOVA (genotype × time) showed a significant effect of genotype (F(2, 33) = 16.88, p < 0.01) and time (F(17, 561) = 471.42, p < 0 .01). The interaction between genotype and time was also significant (F(34, 561) = 7.88, p < 0.01). Bonferroni’s post-hoc test revealed that 3MST-KO rats had significantly higher locomotor activity compared to wild-type rats from 10 min after the MK-801 administration onwards. **b**. Comparison of the total distance traveled by wild-type, 3MST-KO, and TRPA1-KO rats. ANOVA showed that there were significant differences between the groups in the total distance traveled after the injection of MK-801 (F(2,35) = 20.76, p < 0.01) but not before the injection (F(2,35) = 1.13, p = 0.33). Bonferroni's posthoc test showed that the total distance traveled was significantly greater in 3MST-KO rats than in the wild-type and TRPA1-KO rats (p < 0.01). *p < 0.05, **p < 0.01, significantly different as indicated. All data shown are means ± SEM.

Hypersensitivity to NMDA antagonists is associated with the positive symptoms of schizophrenia^52^. We examined the hyperlocomotion induced by the acute administration of MK-801 to 3MST-KO and TRPA1-KO rats, and their responses were compared to those induced in wild-type rats. MK-801 induced hyperlocomotion in all three groups. However, 3MST-KO rats showed a significant increase in locomotor activity after MK-801 administration compared with the other two groups (Fig. 8a,b). Bonferroni’s post hoc tests revealed that the distance traveled by 3MST-KO rats was significantly greater than that by the wild-type rats 10 min after MK-801 injection, and this trend persisted until the end of the measurement (Fig. 8a). In contrast, TRPA1-KO rats showed a significant decrease in locomotion 15 min after MK-801 injection (p < 0.05). Wild-type and KO rats showed equivalent locomotor activity 30 min before the injection of MK-801 (Fig. 8b).

The involvement of the 3MST and TRPA1 channels in working memory was examined using the Y-maze. The total arm entry of both 3MST-KO and TRPA1-KO rats was significantly lower than that of wild-type rats (Supplementary Fig. 8b). However, the percentage of spontaneous alternation in 3MST-KO rats showed a decreasing tendency but was not significantly lower than that in wild-type rats (Supplementary Fig. 8a). The other behavioral tests, open field, pre-pulse inhibition (PPI) and contextual fear conditioning tests, did not show any significant differences between the genotypes (Supplementary Fig. 8c–h).

## Discussion

Since the identification of H_2_S_n_, which activate TRPA1 channels^18,22,23^ and suppress the activity of phosphatase and tensin homolog (PTEN)^53^, most of the previously reported effects of H_2_S have been thought to be mediated by H_2_S_n_.

The target molecules of H_2_S and H_2_S_2_ for regulating transmitter release are localized to the extracellular side of synapses or glia, where cysteine residues can be in oxidized forms, such as cysteine disulfide bonds and nitrosylated or sulfinated cysteine residues. They can be S-sulfurated by H_2_S more efficiently than with H_2_S_2_. Since the intracellular concentrations of H_2_S are greater than those of H_2_S_2_ (Fig. 2c,d), H_2_S may regulate the release of these transmitters to a greater extent than H_2_S_2_.

Since the effect of H_2_S on neurotransmitter release during in vivo microdialysis was investigated using high concentrations of Na_2_S and Na_2_S_2_ (Figs. 3, 4), the observed effect of Na_2_S can be induced by the polysulfides produced by the oxidation of Na_2_S and generated by the subsequent interaction of the produced polysulfide^18,53^. However, this is unlikely because the effect of Na_2_S on the release of GABA and glutamate is greater than that of Na_2_S_2_, which can S-sulfurate cysteine residues, similar to other polysulfides (Fig. 3).

The expected concentrations in the target region of in vivo microdialysis in the present study may not be far from the physiological concentrations or at least less than the toxic levels. Exposure of rats to high levels of H_2_S during perinatal development significantly depressed neurotransmitters^27^, and sublethal doses of NaHS applied to rodents suppressed neurotransmitters^28^. These observations suggest that toxic concentrations of H_2_S suppress neurotransmitter levels.

The release of d-serine, l-serine, and glutamine induced by H_2_S and H_2_S_2_ was not concentration–dependent and less efficient than that of GABA, glutamate, and glycine (Figs. 3, 4), probably because the release mechanism of one group was different from that of the other.

**Figure 9:**
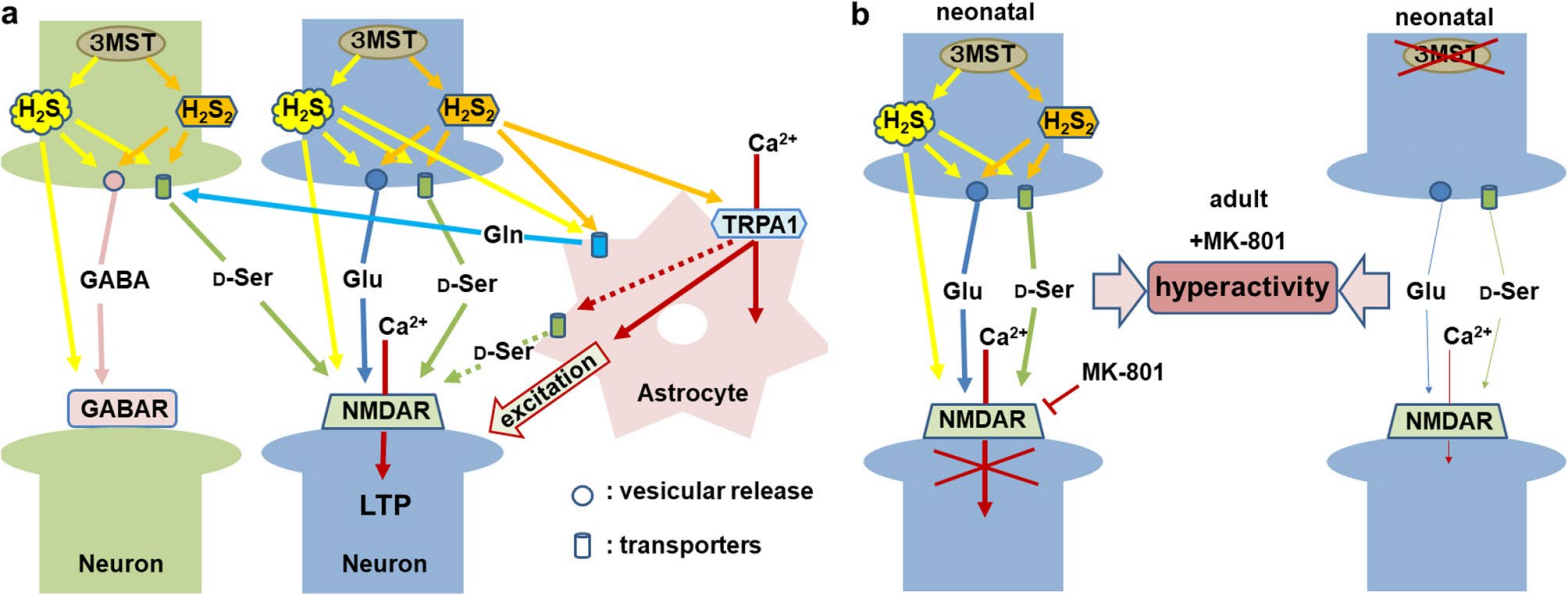
Schematic representation of the possible mechanisms of H_2_S and H_2_S_2_ in LTP and behavioral hyperactivity. **a**. The possible mechanisms of H_2_S and H_2_S_2_ in LTP. H_2_S and H_2_S_2_ produced by 3MST facilitate the release of glutamate and GABA. H_2_S_2_ activates TRPA1 channels to induce Ca^2+^ influx in astrocytes which excite nearby neurons to induce LTP. Glutamine released from astrocytes enhances the release of d-serine. Glutamate and d-serine activate NMDA receptors to induce LTP. **b**. The possible mechanisms for the behavioral hyperactivity induced in 3MST-KO rats. The chronic inactivation of NMDA receptors during the neonatal period by applying MK-801 affects adult behavioral properties. The release of glutamate and d-serine enhanced by H_2_S and H_2_S_2_ is decreased in 3MST-KO, suppressing the activity of NMDA receptors. Suppression of NMDA receptor activity induces hyperlocomotion in 3MST-KO, similar to the animals treated as the antagonist of NMDA receptors in neonatal periods.

H_2_S and H_2_S_2_ showed similar neuroprotective effects against oxidative stress by increasing the intracellular concentrations of glutathione^54^ by activating the cystine/glutamate antiporter and cysteine transporter^54–56^. Alanine-serine-cysteine transporter 1 (Asc-1), a neutral amino acid transporter located in the neurons, mediates the transport of d-serine, glycine, and l-serine^57,58^. Another transporter system, Slc38a1, which is localized to GABAergic neurons in the hippocampus, is also involved in the release of d-serine^59^. These transporters are candidate molecules releasing d-serine, l-serine, and glutamine induced by H_2_S and H_2_S_2_ (Fig. 9a).

d-serine release is enhanced by glutamine, whereas it is suppressed by glycine^39,59–61^. The release of d-serine was not in a concentration of H_2_S and H_2_S_2_ dependent manner, probably due to the suppressive effect of glycine, which is released more readily than glutamine (Figs. 3d,g, 4a,b)^60^.

The release of GABA and glutamate by H_2_S and H_2_S_2_ must be clarified to determine whether H_2_S/H_2_S_2_ excites neurons or stimulates the presynaptic vesicular machinery. Some neurotransmitters regulate the release of H_2_S and polysulfides (Fig. 5), suggesting that they can directly or indirectly activate 3MST. Malfunction of the serotonergic system, which regulates the release of H_2_S, can result in various pathological conditions, including schizophrenia^62^. Understanding the physiological roles of H_2_S and polysulfides both upstream and downstream of these neurotransmitters as well as the regulation of 3MST activity in the nervous system is essential.

Glutamatergic pyramidal neurons and GABAergic interneurons contain SR that release d-serine. GABAergic neurons activate GABA_A_ receptors on astrocytes, which contain higher concentrations of Cl^−^ ions in the cytoplasm to depolarize the membrane^63^, release d-serine^53^, and stimulate nearby neurons to release d-serine.

The present study agrees with a previous study^25^ in that LTP requires TRPA1 activity (Fig. 7e,f), suggesting that astrocytes with activated TRPA1 channels may influence the activity of neighboring neurons^64^, leading to LTP induction (Fig. 9a). The sensitivity of NMDA receptors may be altered depending on the age of the animals, owing to changes in the subunits of the receptors^65^. Therefore, the sensitivity of NMDA observed in neuronal cultures may differ from that observed in adult rats examined for LTP induction. However, because LTP induction was investigated using different genotypes of animals in the same age group, the influence may be small.

The present study showed that MK-801 application to 3MST-KO rats significantly increased hyperlocomotion (Fig. 8). NMDA antagonists induce a behavioral phenotype that resembles the symptoms of schizophrenia, including cognitive impairments^52,66^. Chronic inactivation of NMDA receptors during the neonatal period affects behavioral properties later in life. For example, deleting NR1, a subunit of the NMDA receptor, in corticolimbic interneurons in the early postnatal period augments MK-801-induced hyperlocomotion in adulthood^42^. Since the release of H_2_S and H_2_S_2_ was decreased in 3MST-KO rats (Fig. 2c,d), the activity of NMDA receptors may be chronically reduced (Fig. 9b). This mechanism may be involved in the increase in hyperactivity induced by the acute application of MK-801 in 3MST-KO rats.

The endogenous levels of H_2_S and polysulfides in TRPA1-KO rats were as low as those in 3MST-KO rats because of secondary effects caused by the low levels of 3MST and cysteine in TRPA1-KO rats (Fig. 2a,c–f). Since cysteine is a substrate of CAT, whose activity is regulated by Ca^2+^ to produce 3MP^67^, the activity of TRPA1 channels can regulate cysteine levels. In contrast, cysteine levels in 3MST/TRPA1-double-KO rats were reinstated (Fig. 2a), probably because the accumulated 3MP in the absence of 3MST may induce feedback inhibition of CAT activity. It is necessary to clarify the mechanism of the regulation by TRPA1 channels of the transcription or translation of 3MST as well as the metabolism of cysteine.

The 3MST and TRPA1 channels play critical role in the induction of LTP, whereas only 3MST is involved in behavioral hyperactivity (Figs. 7, 8). TRPA1-KO rats had endogenous levels of H_2_S/H_2_S_2_ as low as those of 3MST-KO rats; however, TRPA1-KO rats still had low levels of 3MST (Fig. 1), which can release the newly synthesized H_2_S and polysulfides. These differences may differentiate the behavioral hyperactivity between 3MST-KO and TRPA1-KO rats.

In conclusion, H_2_S and polysulfides regulate the release of several neurotransmitters, some of which control the release of H_2_S and polysulfides. H_2_S/H_2_S_2_ produced by 3MST is involved in hyperactivity and, together with TRPA1 channels, regulate LTP induction. These molecules, their enzymes, and target molecules have therapeutic potential in psychiatric diseases and cognitive decline.

## Methods

### Chemicals

All methods were performed in accordance with the guidelines and regulations of chemical substance management and approved by the committees of chemical substance management in the Sanyo-Onoda City University, Musashino University and National Institute of Neuroscience, National Center of Neurology and Psychiatry. Na_2_S_2_ (Dojindo, Kumamoto, Japan), Na_2_S_3_ (Dojindo), Na_2_S (Wako pure chemicals, Osaka, Japan), l-cysteine (Wako), and glutathione (Wako) were dissolved at 0.1 M in ultrapure water. These stock solutions were stored at − 80 °C, and were used within a week. 5,7-Dichlorokynurenic acid (DCKA) was obtained from Tocris Bioscience (Avonmouth, UK). d-Serine and the other chemicals were from Wako Pure Chemical Industries, Ltd. (Osaka, Japan).

### Preparation of Cas9 and gRNAs

All the animal experiments in this study were approved by the Animal Research Committee of Sanyo-Onoda City University (Permission number: 2022-1-GA), All experiments were approved and conformed to the guidelines set by the Small Animal Welfare Committee of the National Institute of Neuroscience, National Center of Neurology and Psychiatry (Permission number: 2021032R1), the Animal Research Committee of Osaka University (24-006-042) and the Animal Research Committee of Musashino University (07-A-2022) and performed in accordance with Japan’s and the university’s guidelines. The study is reported in accordance with ARRIVE guideline. All experiments were approved by the DNA Biosafety Committee of Musashino University (Permission number. ExR04-07), Osaka University (03947), Sanyo-Onoda City University (2019-1-GA), the National Institute of Neuroscience, National Center of Neurology and Psychiatry (R4-14) and performed in accordance with Japan’s and the university’s guidelines.

Cas9 mRNA was transcribed in vitro using the mMESSAGE mMACHINE T7 Ultra Kit (Thermo Fisher) from a linearized plasmid (ID #72602; http://www.addgene.org/CRISPR) and purified using a MEGAClear kit (Thermo Fisher). To design gRNAs, software tool (http://www.crispr.genome-engineering.org) predicting unique target sites throughout the rat genome were used. gRNAs were transcribed in vitro using a MEGAshortscript T7 Transcription Kit (Life Technologies) from synthetic double-strand DNAs obtained from IDT (Integrated DNA Technologies, IA, USA) or Life Technologies.

### Electroporation into rat embryos

F344/Jcl (CLEA Japan, Inc. Tokyo, Japan) rat embryos were collected from 7 to 8 weeks of age females that were superovulated by administration of 150 U/kg of PMSG followed by 75 U/kg of HCG. After natural mating, pronuclear-stage embryos were collected from oviducts of the females and cultured in a modified Krebs–Ringer bicarbonate medium (ARK Resource, Kumamoto, Japan). For electroporation, 50–100 embryos 3–4 h after collecting were placed into the chamber with 40 µl of serum free media (Opti-MEM Thermo Fisher Scientific MA, USA) containing 400 ng/µl Cas9 mRNA, 200 ng/µl gRNA. They were electroporated with a 5 mm gap electrode (CUY505P5 or CUY520P5 Nepa Gene, Chiba, Japan) in a NEPA21 Super Electroporator (Nepa Gene, Chiba, Japan). The poring pulses for the electroporation were voltage 225 V, pulse width 2.0 ms for rat embryos, pulse interval 50 ms, and number of pulses + 4. The first and second transfer pulses were voltage 20 V, pulse width 50 ms, pulse interval 50 ms, and number of pulses + 5. Embryos that developed to the two-cell stage after the introduction of RNAs were transferred into the oviducts of female surrogates anesthetized with isoflurane (DS Pharma Animal Health Co., Ltd., Osaka, Japan).

### Genotyping analysis

Genomic DNA was extracted from the tail tip using the KAPA Express Extract DNA Extraction Kit (Kapa Biosystems, London, UK). For PCR and sequence analysis, we used specific primers which could amplify the targeted region. PCR was performed in a total volume of 15 μl under the following conditions; 1 cycle of 94 °C for 1 min, 30 cycles of 98 °C for 10 s, 60 °C for 15 s and 68 °C for 30 s. The final reaction mixture contained 200 μM dNTPs, 1.0 mM MgCl_2_, and 0.66 μM of primer. The PCR products were then directly sequenced using the BigDye Terminator v3.1 cycle sequencing mix and the standard protocol for an Applied Biosystems 3130 DNA Sequencer (Life Technologies).

### Generation of 3MST- and TRPA1-deficient rats

Each founder rat harboring the 3MST mutant allele in exon 2 or the TRPA1 mutant allele in exon 1 was crossed with wild-type rats to obtain 3MST- or TRPA1-heterozygous rats. The heterozymous rats were crossed to obtain homozygous rats. Genotyping was performed by means of PCR and determined by sequencing. The genotyping primers were 5′-TGGTATCTTTCCTGTCTTGCAG-3′ and 5′-CGAAATGCGTGGCACTAGG-3′ for 3MST, and 5′-CAGAACCGGCTTTAGCTTCA-3′ and 5′-GCCGTGCTTCCTAAACTTGA-3′ for TRPA1.

### Histology

For histological analysis, male rats were anesthetized with a mixture of medetomidine (0.375 mg/kg), midazolam (2.0 mg/kg), and butorphanol (2.5 mg/kg) and perfused with 0.02 M phosphate buffered saline (PBS) followed by 4% paraformaldehyde (PFA). Then the organs (brain, lung, and kidney) were quickly removed and post-fixed in 4% PFA for more than 24 h. These samples were cryoprotected with 30% sucrose PFA, then quickly frozen with dry ice-hexane cooling bath. Samples were cut using microtome (Yamato Kohki, Osaka, Japan) and 8-µm sections were collected. For the brain, sagittal and coronal sections were obtained. These sections were mounted on glass slides and air-dried before staining. Coronal sections of the brain containing the dorsal hippocampus were stained with cresyl violet. For the remaining sections, hematoxylin–eosin (HE) staining was conducted. Photographs of each section were obtained using a microscope (Olympus) equipped a digital camera (Wraymer, Osaka, Japan) connected to a computer.

### Preparation of brain tissue for LC–MS/MS and HPLC analysis

For LC–MS/MS analysis of H_2_S_n_ and cysteine persulfide, brain extracts were prepared according to the previously reported method with some modification^12^. Briefly, hippocampus removed from 30 day old male F344 rats, was homogenized with 9 volume of ice-cold homogenizing buffer (10 mM phosphate buffer pH7.0 containing 1% Triton X-100, EDTA-free complete as protease inhibitor, 0.1 mM diethylenetriamine-N,N,N′,N″,N″-pentaacetic acid (DTPA) and 2 mM monobromobimane) and centrifuged at 12,000×*g* for 10 min at 4 °C. Supernatant was transferred to a new tube and left on bench for 30 min in dark at room temperature to label thiol residues of H_2_S_n_ and cysteine persulfide. Reaction was stopped by adding 5-sulfosalicyclic acid (SSA: final concentration 2%) and incubated for 15 min on ice. The reaction mixture was centrifuged at 12,000×*g* for 10 min, supernatant was analyzed with LC–MS/MS (Agilent 6470 Triple Quad LC/MS, Santa Clara, USA).

For HPLC analysis of amino acid, brain extracts were prepared according to the previously reported method with minor modification^68^. Briefly, hippocampus was homogenized with 5 volume of methanol at 1500 rpm for 10 strokes, left on bench for 10 min, centrifuged at 10,000×*g* for 10 min at 4 °C. Supernatant was analyzed with HPLC (Waters 2695, Milford, USA).

### Suspensions of brain cells

The suspensions of brain cells were prepared by the modified method reported previously^69^. Briefly, brains of male F344 rats were removed at the postnatal day 7–14. After meninges were removed, brains were chopped to approximately 1 mm cubes with scissors in the basic medium containing 3 mg/ml BSA fraction V (Sigma-Aldrich, St. Louis, MO, USA), 14 mM glucose (Sigma), 1.2 mM MgSO_4_ in Ca^2+^ free HBSS (Wako pure chemicals, Osaka, Japan). The suspended brain cubes were centrifuged at 100×*g*, 4 °C for 20 s to remove supernatant, and washed once with the basic medium. The brain cubes were incubated in 10 ml basic medium containing 0.025% trypsin EDTA (Wako) for 15 min at 37 °C, and then 10 ml basic medium containing 6.4 μg/ml DNase I (Sigma-Aldrich), 0.04 mg/ml Soybean Trypsin Inhibitor (SBTI) (Sigma-Aldrich) was added and gently mixed. The supernatant was removed after centrifugation at 100×*g* for 20 s at room temperature. Two ml basic medium containing 40 μg/ml DNase I, 0.25 mg/ml SBTI, and 3 mM MgSO_4_ was added to the brain cubes and mixed gently up and down with a pipette without making foams for 30 times. After centrifugation at 210×*g* for 1 min, cells were recovered and washed with 2 ml HBSS with Ca^2+^ and Mg^2+^ medium (Wako) containing 14 mM glucose (Sigma-Aldrich) for 3 times, and then preincubated at 37 °C for 1 h in a shaker at 100 rpm (Taitec Bio-shaker BR-40LF, Saitama, Japan) before used for experiments.

### Measurement of endogenous polysulfides and neurotransmitters and those released from brain cell suspension

After 1 h preincubation, 500 μl suspensions of brain cells were incubated for 15 min at 37 °C in the presence of 200 μM neurotransmitters (Sigma-Aldrich), centrifuged at 1000×*g* for 1 min to separate supernatant (cell-sup) and pellet. Pellets were homogenized in homogenizing buffer and centrifuged at 12,000×*g* for 10 min at 4 °C to recover supernatant (pellet-sup).

Thiol residues of Na_2_S_n_ and cysteine persulfides in cell-sup and pellet-sup were labelled with monobromobimane as described above and analyzed by LC–MS/MS.

### Measurement of transmitters released and changes in their endogenous levels in cells

After preincubation, brain cell suspensions were incubated for 15 min at 37 °C in the presence of 20 μM Na_2_S, Na_2_S_2_ and Na_2_S_3_. Na_2_S_n_ (n = 1–3) were dissolved and diluted in water until 100 times of the final concentrations to prevent the production of S8 precipitates, then dissolved into the corresponding buffer solution. After the exposure to Na_2_S_n_ the suspensions of brain cells were centrifuged to separate supernatant (cell-sup) and pellets. Pellets were homogenized with methanol and centrifuged at 10,000×*g* for 10 min at 4 °C to separate supernatant (pellet-sup). Cell-sup and pellet-sup were analyzed with HPLC.

### HPLC analysis

For HPLC measurement of amino acids including d-serine the method by Grant et al.^70^ was employed. Automated pre-column derivatization was carried out by drawing up a 5 µl aliquot of sample solution and 5 µl of derivatizing reagent solution consisting of 1 mg o-phthaldialdehyde and 2 mg N-isobutyryl-l-cysteine in 0.1 ml methanol and 0.9 ml 0.2 M sodium borate buffer (pH 10), then holding in the injection loop 5 min prior to injection to the HPLC (Waters alliance 2695). Amino acids were separated by a Symmetry C18 column (4.6 mm × 150 mm, 3.5 µm). The flow rate was 0.7 ml/min and run time was 85 min. Solvent A comprised 850 ml 0.04 M sodium phosphate and 150 ml methanol (pH 6.2). Solvent B comprised 670 ml 0.04 M sodium phosphate, 555 ml methanol, and 30 ml tetrahydrofuran, adjusted to pH 6.2. The amino acids were separated by a concave gradient from 15 to 100% B in 50 min. The solvent mix was returned to initial conditions by 65 min using a concave gradient and maintained at that composition for 20 min prior to the next injection. Amino acid derivatives were monitored at excitation and emission wavelengths of 260 and 455 nm, respectively (Water 2475 fluorescence detector).

### In vivo microdialysis

Prior to the experiments, male F344 rats at 7–8 weeks old were implanted with microdialysis guide cannula targeting the dorsal hippocampus under the same anesthesia as described in Histology. After surgery, animals were allowed to recover for at least 24 h before experiments. At the start of experiments, the dialysis probes (A-I-3-01; EICOM, Kyoto, Japan) with 1.0 mm-long membranes were inserted into the guide cannula and were continuously perfused with perfusion medium (150 mM NaCl, 2.2 mM CaCl_2_, 4.0 mM KCl) at a flux rate of 2.0 µl/min using a syringe pump (ESP-64; EICOM). Each rat was attached to a swivel unit to allow free movement. After 60 min equilibration period, perfusates were collected every 20 min. Following 60 min baseline sampling, Na_2_S_2_ or Na_2_S (1 mM, 3 mM, and 10 mM) dissolved in perfusion medium were perfused for 20 min at 40 min intervals in ascending order. After 100 min of additional sampling, rats were sacrificed under isoflurane anesthesia and the location of the dialysis probes was verified in 40 μm-thick brain slices.

High-performance liquid chromatography with an electrochemical detector (HTEC-700, EICOM, EICOM, Kyoto, Japan) was used for quantification of amino acids in micridialysis experiments. For quantification of d-serine, l-serine, glutamate, glutamine, and glycine, an aliquot of each dialysate sample was derivatized with N-acetyl-l-cysteine and o-phthaldialdehyde for 300 s at 10 °C and then analyzed on a reverse-phase column (Eicompak EX-3ODS, 4.6 φ × 100 mm, EICOM) operated at the constant flow rate of 0.5 ml/min at 30 °C. The potential of the glassy carbon electrode (WE-GC, EICOM) was set at + 0.6 V (vs. Ag/AgCl). Composition of the mobile phase for the measurement was 100 mM phosphate buffer (pH 6.0), methanol (18 v/v %) and 5 mg/L EDTA·2Na. For quantification of GABA, an aliquot of each dialysate sample was derivatized with 2-mercaptoethanol and o-phthaldialdehyde for 150 s at 10 °C and then analyzed on a reverse-phase column (Eicompak FA-30DS, 3 φ × 75 mm, EICOM) operated at the constant flow rate of 0.5 ml/min at 40 °C. The potential of the glassy carbon electrode (WE-GC, EICOM) was set at + 0.6 V (vs. Ag/AgCl). Composition of the mobile phase for the measurement was 100 mM phosphate buffer (pH 6.0), methanol (7 v/v %), acetonitrile (13 v/v %) and 5 mg/L EDTA·2Na.

### Primary cultures of neurons

Brains were removed from postnatal day 3 to 7 of male F344 rats and the hippocampus was dissected in L15 medium (Life Technologies). The tissue was chopped and digested with 0.25% trypsin (Sigma-Aldrich) and 0.1% DNase I (Sigma-Aldrich) in Ca^2+^/Mg^2+^-free PBS for 15 min at 37 °C. After mechanical dissociation, cells were plated onto poly-d-lysine-coated 35 mm dishes (BD Biosciences, San Jose, CA, USA) and cultured in Neurobasal medium (Life Technologies) supplemented with B27 (Life Technologies) for 2 days at 37 °C in 5% CO_2_. Cells were further cultured in the presence of 5 μM cytosine β-d-arabinofuranoside (AraC, Sigma-Aldrich) for 1 day, and washed once with Neurobasal medium supplemented with B27 to remove AraC. Cells cultured for additional 3–4 days were used for experiments.

### Primary cultures of astrocytes

Primary cultures of astrocytes were prepared from samples obtained from male F344 rats at postnatal day 3 to 7. The hippocampus was excised in L15 medium (Life Technologies Corp., Carlsbad, CA, USA), and digested with 0.25% trypsin (Sigma-Aldrich) and 0.1% DNase I (Sigma-Aldrich) in Ca^2+^/Mg^2+^-free PBS for 15 min at 37 °C. The cells were plated at a density of 1600 cells/mm^2^ onto poly-d-lysine-coated 35-mm dishes (Becton, Dickinson and Co., Franklin Lakes, NJ, USA) and incubated in DMEM (Life Technologies) supplemented with 5% fetal bovine serum (FBS; Japan Bio Serum, Hiroshima, Japan), 5% horse serum (Life Technologies), and penicillin/streptomycin (50 U/ml; Sigma-Aldrich) at 37 °C in 5% CO_2_.

### Ca^2+^ imaging of neurons and astrocytes

The cells were washed once with basal salt solution (BSS) consisting of 130 mM NaCl, 5.4 mM KCl, 1.8 mM CaCl_2_, 0.8 mM MgCl_2_, 5.5 mM glucose, and 10 mM HEPES–NaOH (pH 7.3) and were loaded with 1 µM calcium green-1/acetoxymethyl ester (Life Technologies) and 0.01% (v/v) cremophorEL in BSS solution for 50 min at 37 °C. The dishes were mounted on an upright fixed stage microscope (Leica DM-LFS; Leica Microsystems, Wetzlar, Germany) and perfused at 2 ml/min. The images were acquired using a water-immersion objective (X20; 0.5 NA; Leica Microsystems) and a CCD camera (C4742-95-12ER; Hamamatsu Photonics, Shizuoka, Japan). The frame duration ranged from 147 to 207 ms, and each image was acquired at 4 s intervals and 4 X 4 binning. Images were acquired and analyzed using Aquacosmos 2.0 software (Hamamatsu Photonics). Changes in calcium concentrations were monitored as a change in the fluorescence intensity (F) relative to the control image (F0) that was acquired before stimulation.

### Field EPSP recordings and induction of LTP

Preparation of hippocampal slices and recording of evoked potentials were made as described in our previous papers^71^. In brief, 400 μm hippocampal slices were prepared from male wild type (F344), 3MST-KO, TRPA1-KO, and 3MST/TRPA1-double KO rats (5–13 weeks old) and maintained in a chamber at 30 °C, where they were continuously perfused with oxygenated (95% O_2_:5% CO_2_) artificial cerebrospinal fluid (ACSF). The composition of the ACSF was as follows (in mM): 124 NaCl, 1.24 KH_2_PO_4_, 3 KCl, 2.2 CaCl_2_, 1.4 MgSO_4_, 25.0 NaHCO_3_, and 10.0 glucose. Schaffer collaterals were stimulated by a bipolar tungsten electrode placed in the stratum radiatum of the CA1 region near the CA2/CA1 border, and the evoked field EPSPs (fEPSPs) were recorded using an extracellular glass microelectrode filled with 0.9% NaCl (tip resistance 2–8 MΩ) placed in stratum radiatum of the CA1 region. Signals were amplified using Axopatch 200B (Molecular Devices) and digitized with a DigiData 1322A or 1550B (Molecular Devices), and acquired using pClamp software (Molecular Devices). Single-pulse test stimulations (100 μsec duration) were applied at 30-s intervals. The stimulus intensity was adjusted in the range of 25–55 μA to evoke fEPSPs of 50% of the maximum amplitude. To induce LTP, a high-frequency stimulation (100 pulses at 100 Hz) was applied at the same intensity with the test stimulation. The rising slope of fEPSP was measured as an index of synaptic efficacy.

### LC–MS/MS analysis

Samples derivatized with monobromobimane (mBB) (Life Technologies) were analyzed by the triple-quadrupole mass spectrometer coupled to HPLC (Agilent technology, LC–MS/MS 6470). Samples were subjected to a reverse phase Symmetry C18 HPLC column (4.6 × 250 mm, Waters) at the flow rate of 1.0 ml/min. The mobile phase consisted of (A) 0.1% formic acid in water and (B) 0.1% formic acid in methanol. Samples were separated by eluting with a gradient: 5% B at 0–5 min and 5–90% B at 5–25 min. The column oven was maintained at 40 °C. The efuent was subjected to the mass spectrometer using an electrospray ionization (ESI) interface operating in the positive-ion mode. The source temperature was set at 400 °C, and the ion spray voltage was at 4.5 kV. Nitrogen was used as a nebulizer and drying gas. The tandem mass spectrometer was tuned in the multiple reaction monitoring mode to monitor mass transitions in positive ion mode: CysSS-mBB m/z 344 → 192, CysSSS-mBB m/z 376 → 192, mBB-S-mBB m/z 415.3 → 193, mBB-S2-mBB m/z 447.3 → 192, mBB-S3-mBB m/z 479.3 → 192.

### Western blot analysis

Hippocampus was homogenized with 9 volumes of RIPA buffer [50 mM Tris/HCl (pH 7.4), 150 mM NaCl, 1% Triton-X, 0.5% Sodium Deoxycholate, 0.1% SDS, 1 mM EDTA, and protease inhibitor cocktail] using a Potter-type glass homogenizer with a Teflon pestle (1500 rpm, 10 strokes). The homogenates were centrifuged at 1000×*g* for 10 min, and the supernatant was recovered. The protein samples (20 µg) were separated by SDS-PAGE on a polyacrylamide gel and transferred to the Immobilon PVDF membrane (Merck Millipore). The membrane was blocked with PBS-T (PBS with 0.1% Tween 20) containing 2% Difco skim milk (Beckton Dickinson) for 90 min at RT and incubated with anti-3MST (1:2,000; Sigma-Aldrich), or anti-TST (1:20,000; Sigma-Aldrich) overnight at 4 °C. The membrane was washed 3 times with PBS-T and incubated with the secondary anti-rabbit antibodies conjugated with horseradish peroxidase diluted at 1:2,500 (GE Healthcare) for 2 h at RT. The binding of the antibodies was visualized with the ImmunoStar Zeta (Fujifilm Wako) and detected using ChemiDoc touch imaging system (Bio-Rad) with imageJ image processing software.

### Measurement of bound sulfane sulfur

A whole brain homogenates were prepared with 9 volumes of ice-cold buffer consisting of 10 mM potassium phosphate (pH 7.4), 1% TritonX-100, 10 mM hydroxylamine, which was used to suppress the activity of PLP-dependent enzymes involved in enzymatic H_2_S production, and protease inhibitor cocktail “complete” (Roche Diagnostics, Mannheim, Germany) using a Potter type glass homogenizer with a Tefon pestle (1,500 rpm, 10 strokes). To lyse brain cell membranes, homogenates were mixed with vortex for 1 min on ice three times with 10 min intervals. The lysates were centrifuged at 12,000×*g* for 10 min, and the supernatants were recovered. For measurement of H_2_S released from bound sulfane sulfur, the method previously reported was used^7^. Briefy, 0.1 ml of supernatants (2.5 mg protein/ml) mixed with 0.1 ml of 15 mM DTT in 100 mM Tris/HCl (pH 9.0), was placed in a 15 ml centrifugation tube, then sealed and incubated at 37 °C for 50 min. Afer adding 0.4 ml of 1 M sodium citrate bufer, pH 6.0, the mixtures were incubated at 37 °C for 10 min with shaking at 125 rpm on a rotary shaker NR-3 (TAITEC) to facilitate release of bound sulfur as H_2_S gas from the aqueous phase. Two ml of approximate 14.5 ml of head-space gas was applied to a gas chromatograph (GC-14B; Shimazu, Kyoto, Japan) equipped with a fame photometric detector and a data processor C-R8A Chromatopac (Shimazu). A reaction mixture without samples was used as a control for a release of H_2_S from DTT.

### MK-801-induced hyperlocomotion

Preceding to the test, male F344 rats at 7–8 weeks old were handled for 5 min a day for 3 days. In the testing, rats were put into the arena (50 × 50 × 50 cm) made of black polyvinyl chloride and were allowed to move freely. After 30 min of baseline period, MK-801 (Sigma-Aldrich, 0.2 mg/kg) dissolved in saline was administered subcutaneously. Then rats were immediately returned to the arena and tested for an additional 60 min^72^. Rat behavior in the arena was recorded using video camera connected to a computer and distance traveled was measured every 5 min using SMART 3.0 video tracking software (Panlab, Barcelona, Spain).

### Statistical analysis

All the statistical analyses of the data were performed using Microsoft Excel 2023 for Window 10 (Microsoft, Redmond, WA, USA) with the add-in software Analysis Tool Pack. Differences between 2 groups were analyzed with Student’s t test. One-way and two-way ANOVA followed by Bonferroni’s post-hoc test were used for the analysis of hyperlocomotion.

### Supplementary Information

Supplementary Information.

## Data Availability

All data generated during this study are included in this published article and supplementary information files.
