# Supplementary material for: Hydrogen sulfide and polysulfides induce GABA/glutamate/d-serine release, facilitate hippocampal LTP, and regulate behavioral hyperactivity
Source: Sci Rep. 2023 Oct 31;13:17663. doi: 10.1038/s41598-023-44877-y (PMC10618189; doi:10.1038/s41598-023-44877-y)
Supplement: Supplementary file 1 — Supplementary Information. [file 41598_2023_44877_MOESM1_ESM.pdf]

Supplementary information

Hydrogen sulfide and polysulfides induce GABA/glutamate/D-serine release, facilitate hippocampal LTP, and regulate behavioral hyperactivity

Hiroki Furuie[1, #], Yuka Kimura[2, #], Tatsuhiro Akaishi[3, #], Misa Yamada[1], Yoshiki Miyasaka[4], Akiyoshi Saitoh[5], Norihiro Shibuya[2], Akiko Watanabe[6], Naoki Kusunose[7], Tomoji Mashimo[4, 8], Takeo Yoshikawa[6], Mitsuhiko Yamada[1, 9], Kazuho Abe[3], Hideo Kimura[2]*

1. Department of Neuropsychopharmacology, National Institute of Mental Health, National Center of Neurology and Psychiatry, Kodaira, Tokyo, Japan.
2. Department of Pharmacology, Sanyo-Onoda City University, Sanyo-Onoda, Yamaguchi, Japan.
3. Laboratory of Pharmacology, Faculty of Pharmacy and Research Institute of Pharmaceutical Sciences, Musashino University, Nishi-Tokyo, Tokyo, Japan
4. Institute of Experimental Animal Sciences, Departement of Medicine, Osaka University, Suita, Osaka, Japan.
5. Department of Pharmacology, Faculty of Pharmaceutical Sciences, Tokyo University of Science, Noda, Chiba, Japan
6. Laboratory of Molecular Psychiatry, RIKEN Center for Brain Science, Wako, Saitama, Japan.
7. School of Pharmaceutical Sciences, Kyushu University of Health and Welfare, Nobeoka, Miyazaki, Japan.
8. Division of Animal Genetics, Laboratiry Animal Research Center, Institute of Medical Science, The Universtiry of Tokyo, Tokyo, Japan
9. Department of Pathophysiology, Faculty of Human Nutrition, Tokyo Kasei Gakuin University, Chiyoda-ku, Tokyo, Japan.

# Authors contributed equally to this study

＊ Correspondence and material requests should be addressed to Hideo Kimura, e-mail:kimura@rs.socu.ac.jp
ORCID ID: https://orcid.org/0000-0002-6069-0475

WT (3MST +/+)

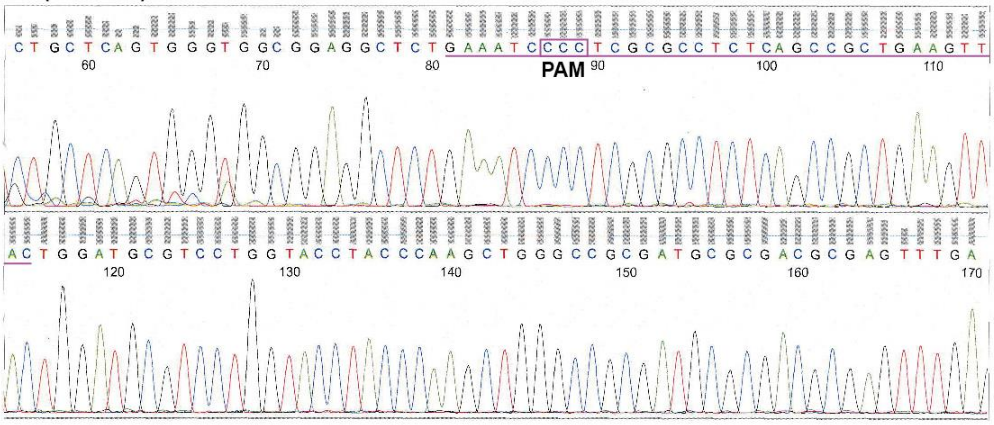

3MST heterozygous (+/-) #5

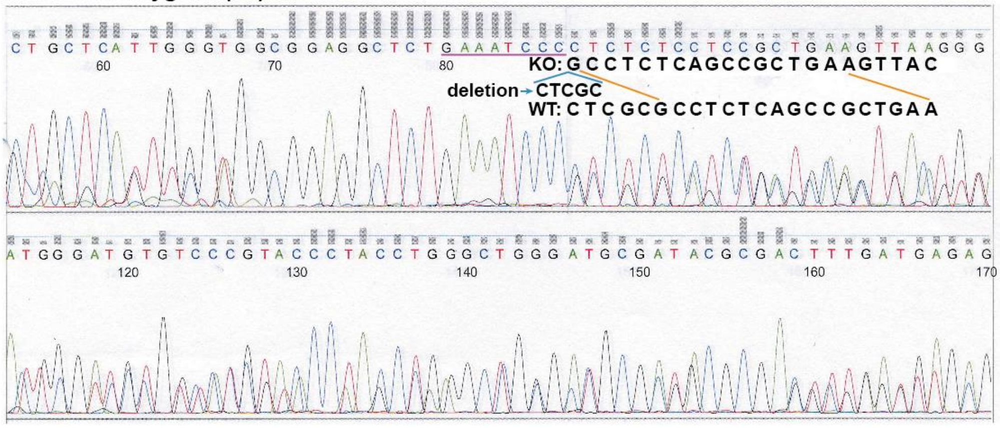

3MST homozygous (-/-) #5

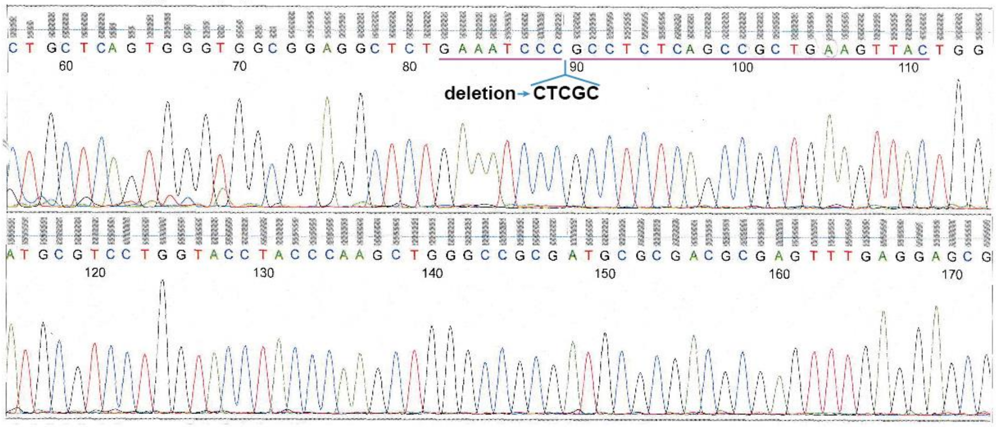

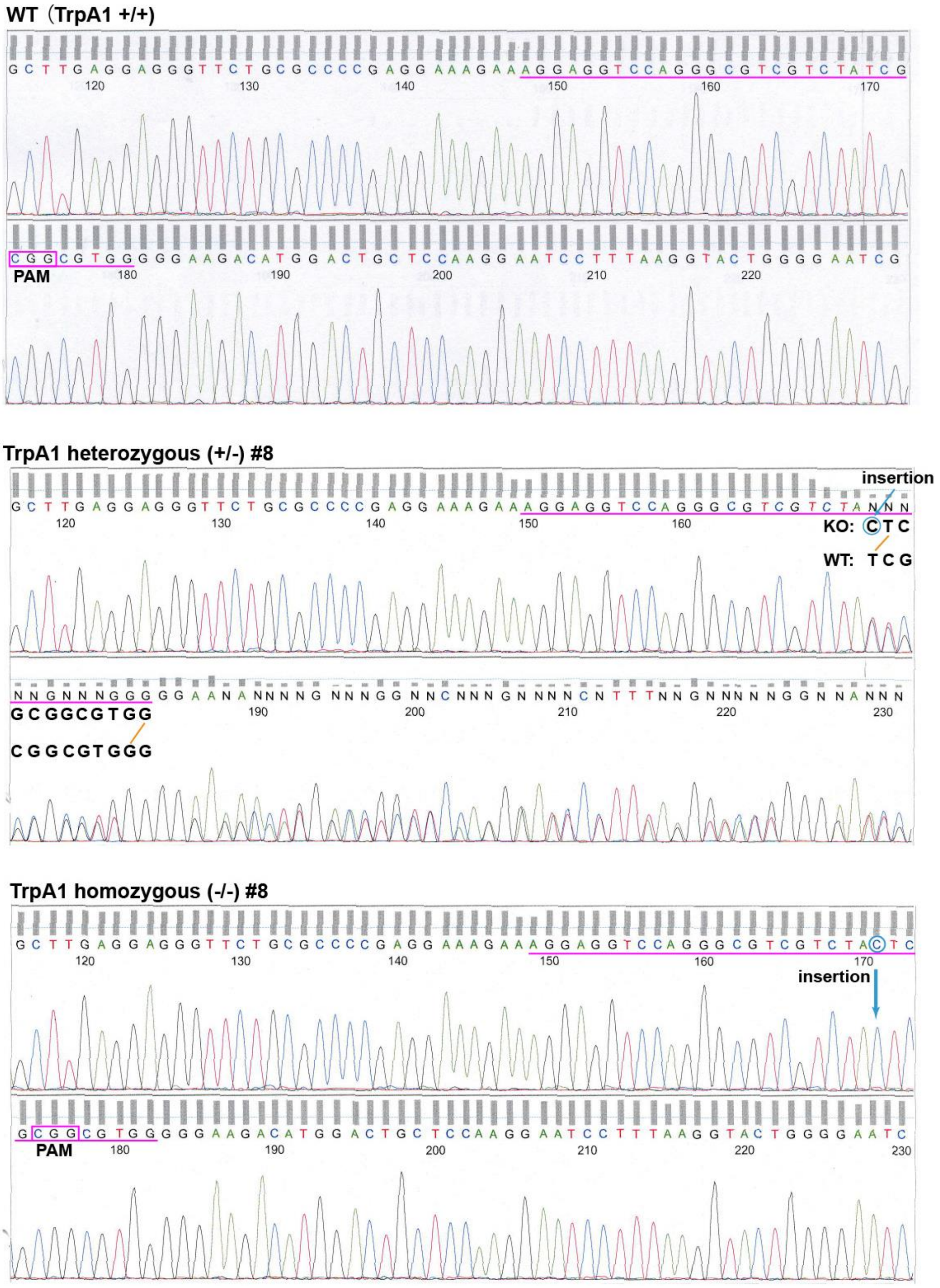

Supplementary figure 1. Genotyping of 3MST and TRPA1 and their knockout. Sequences of 3MST heterozygous and homozygous and TRPA1 channel heterozygous and homozygous were compared with those of the wild-type.

a 3MST

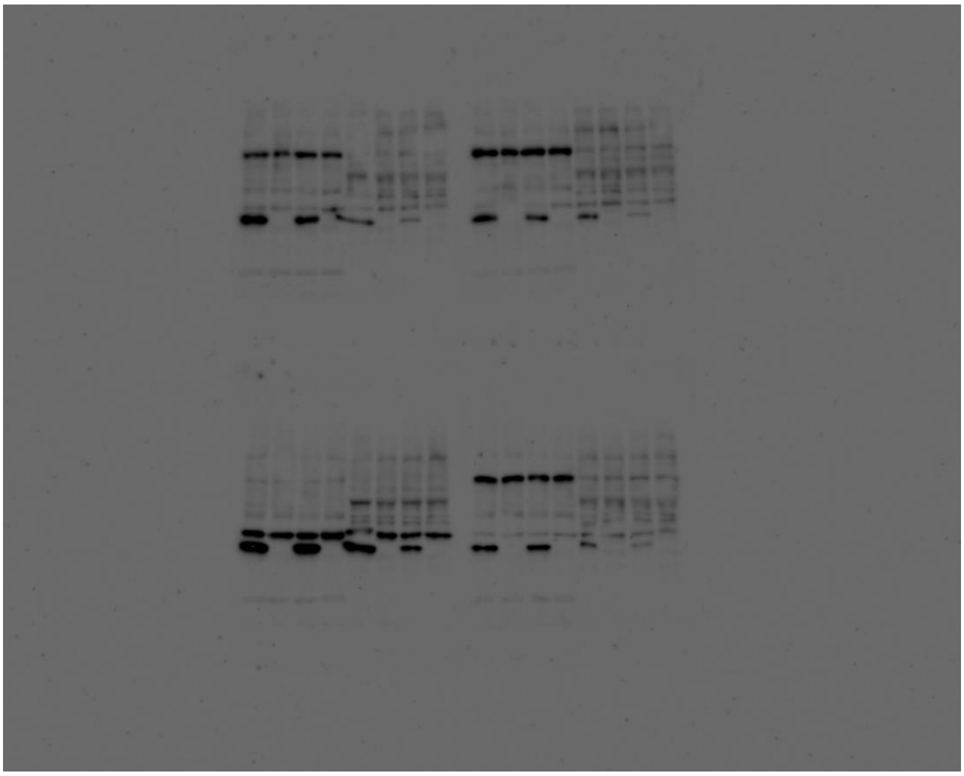

b 3MST

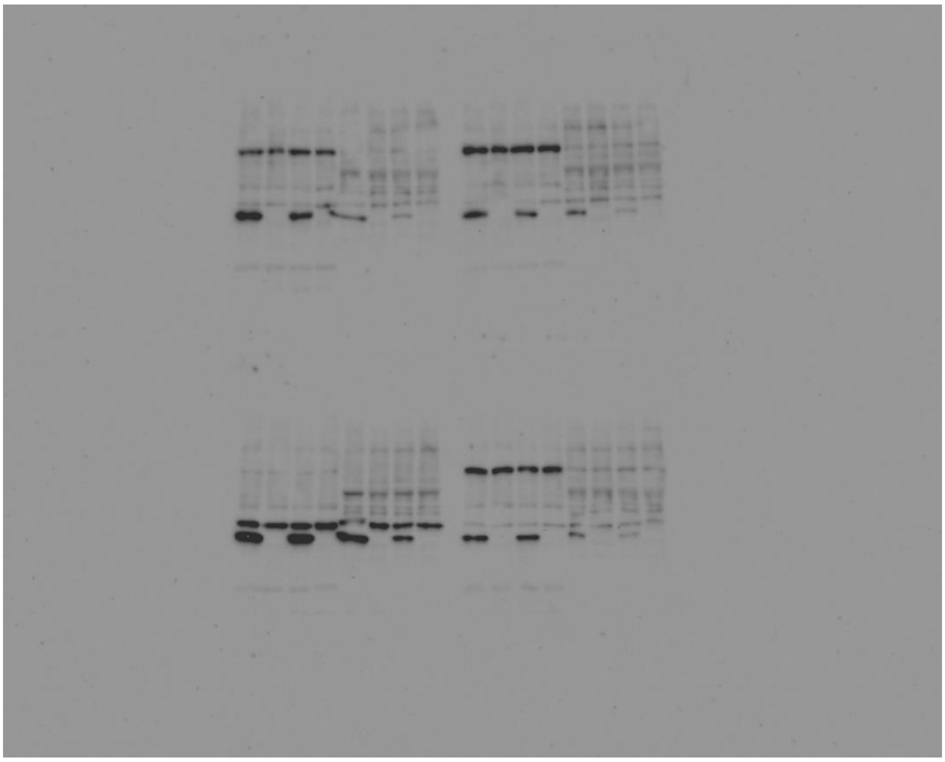

C 3MST

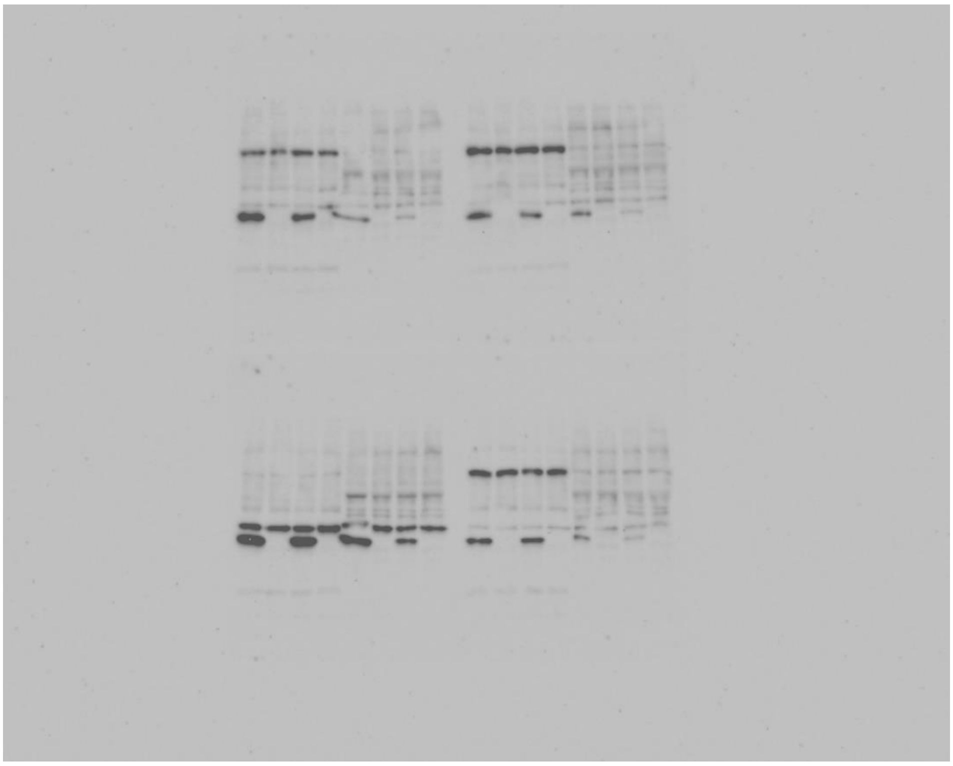

d 3MST

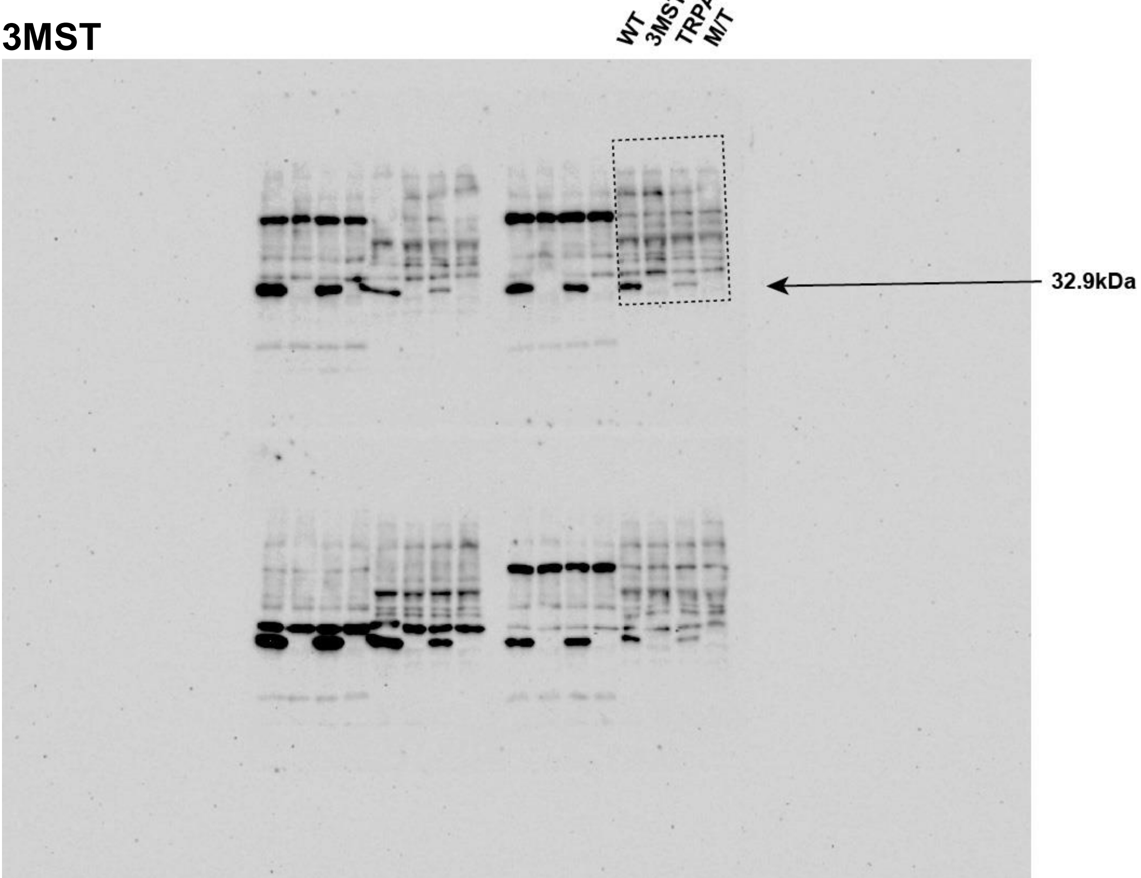

e GAPDH

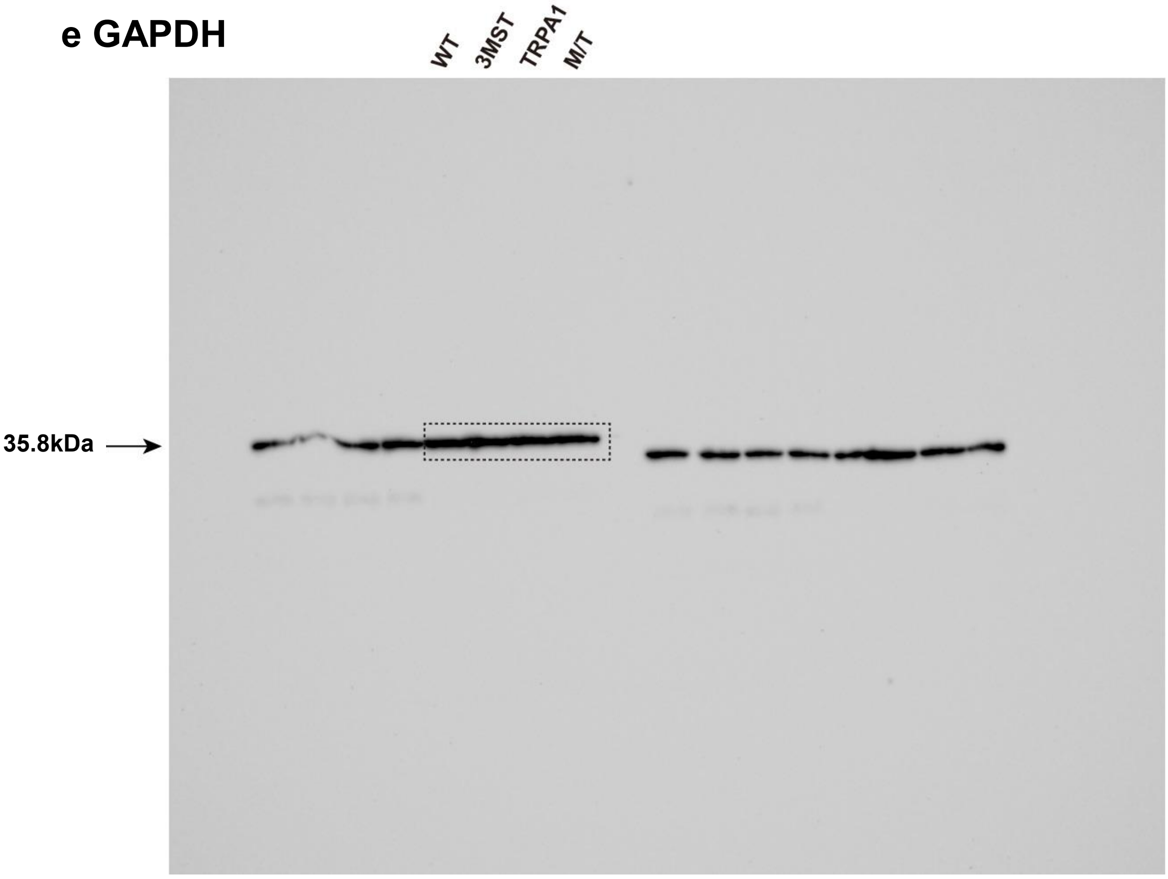

f GAPDH

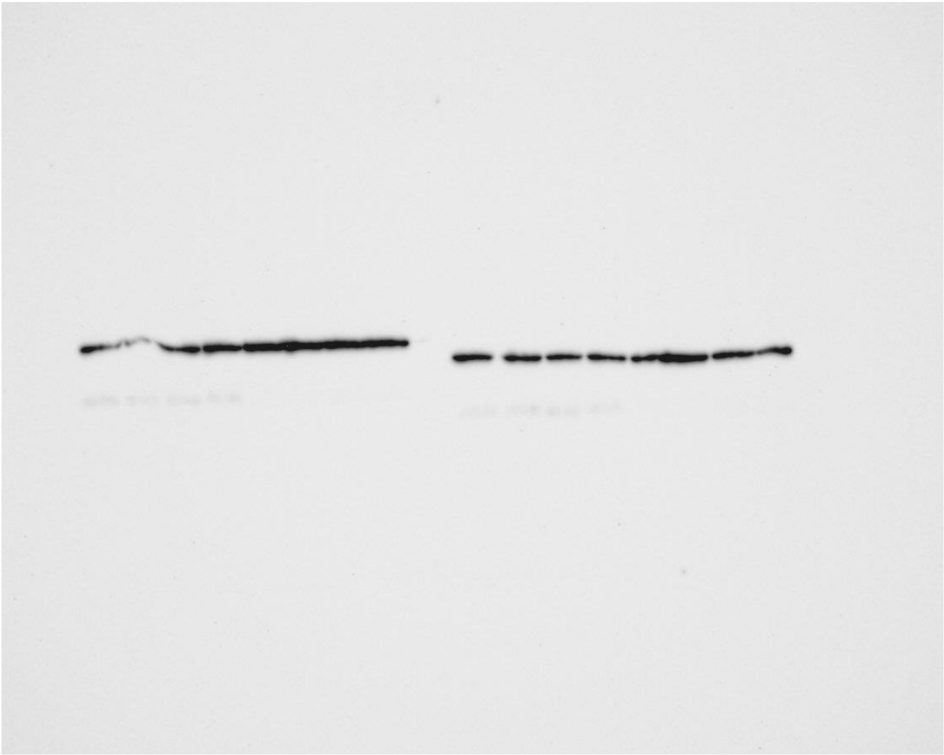

g GAPDH

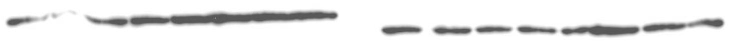

h GAPDH

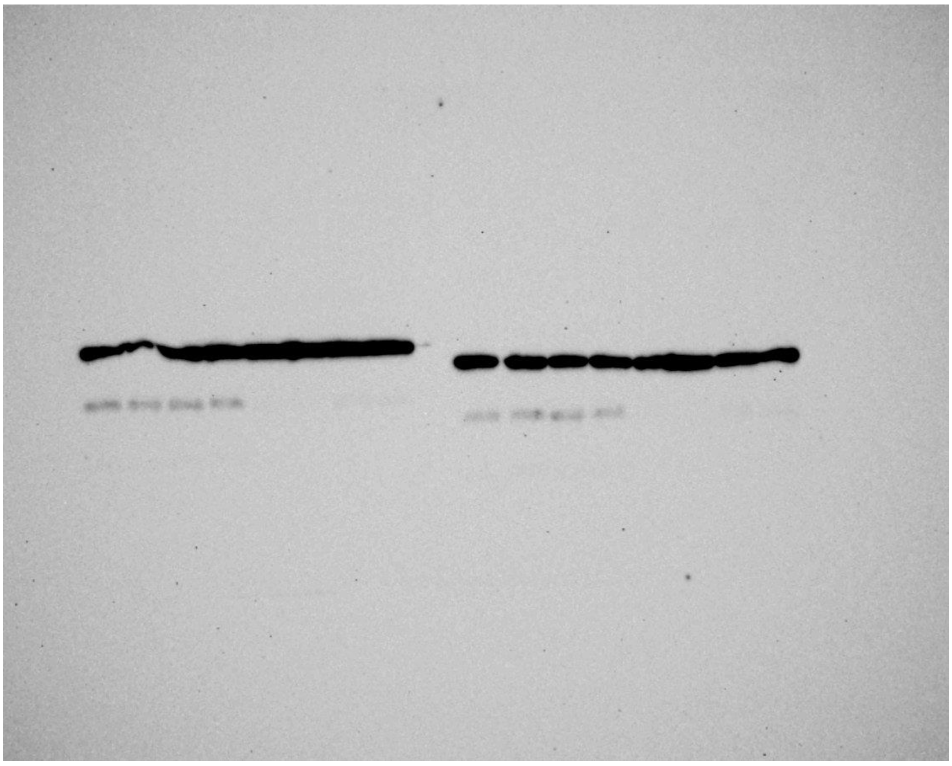

i TST

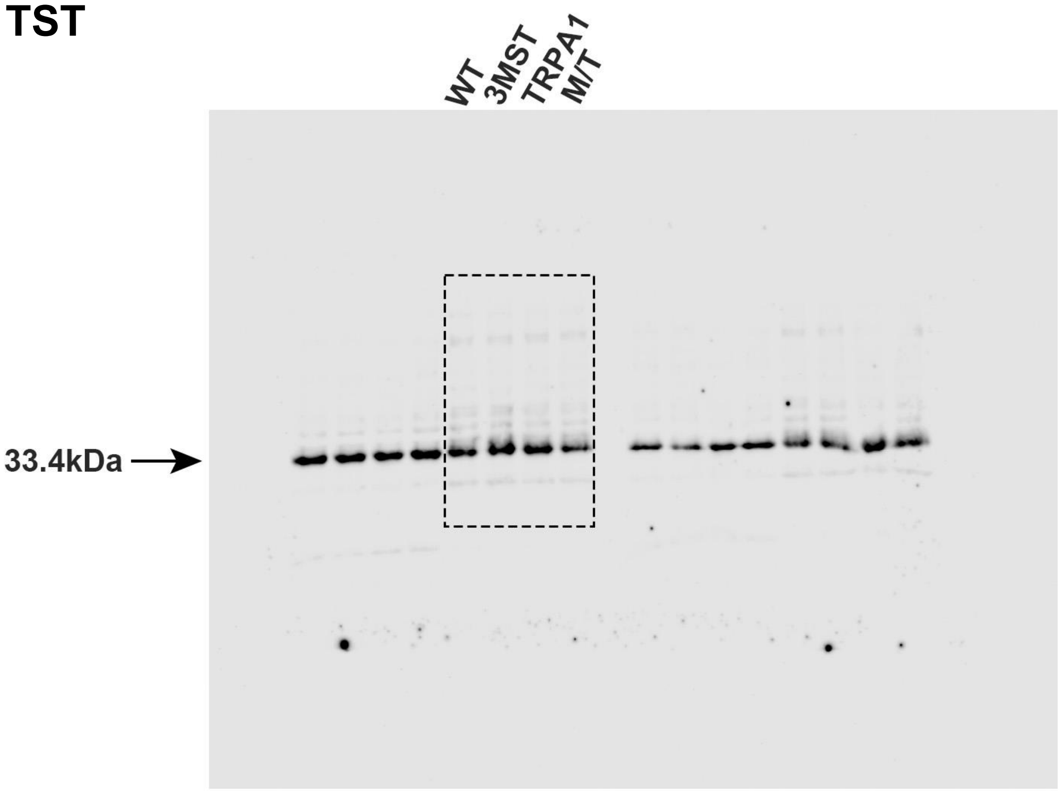

j TST

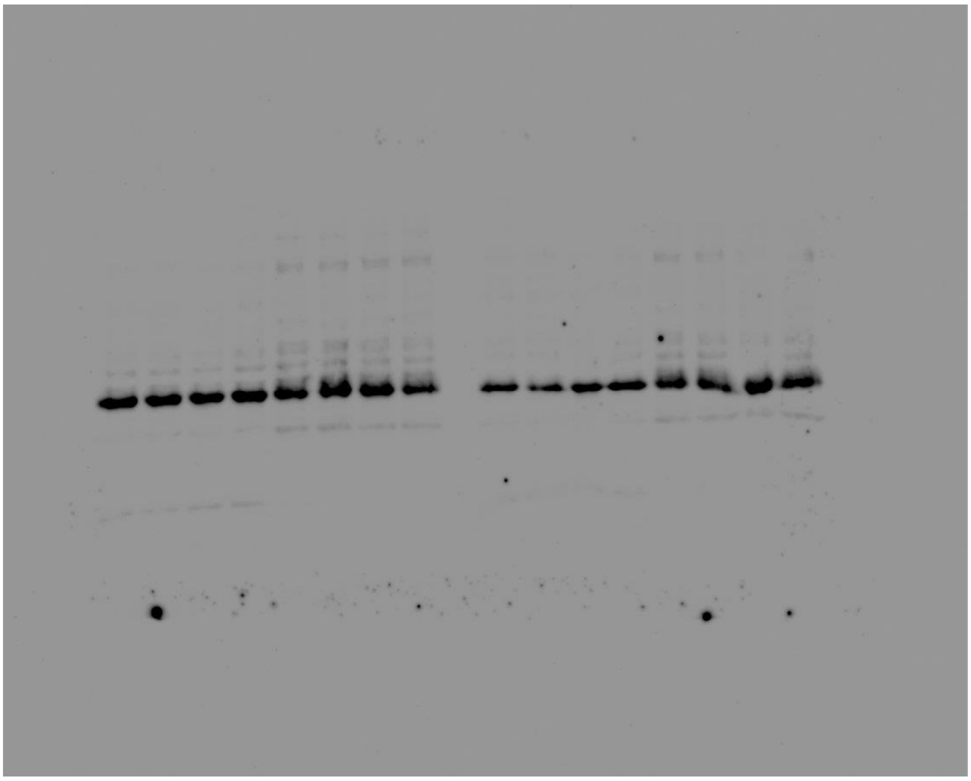

k TST

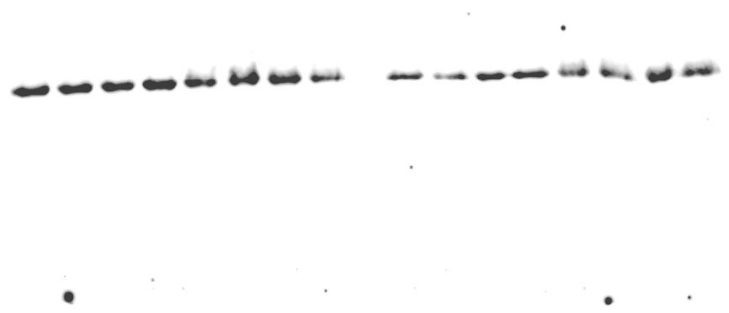

l TST

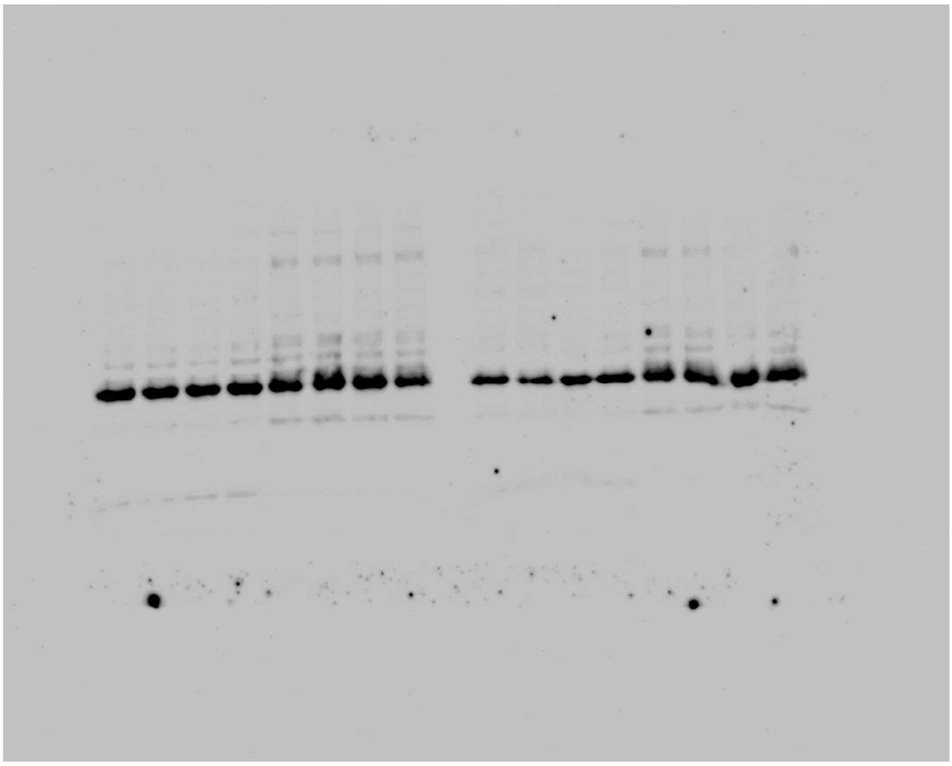

m GAPDH

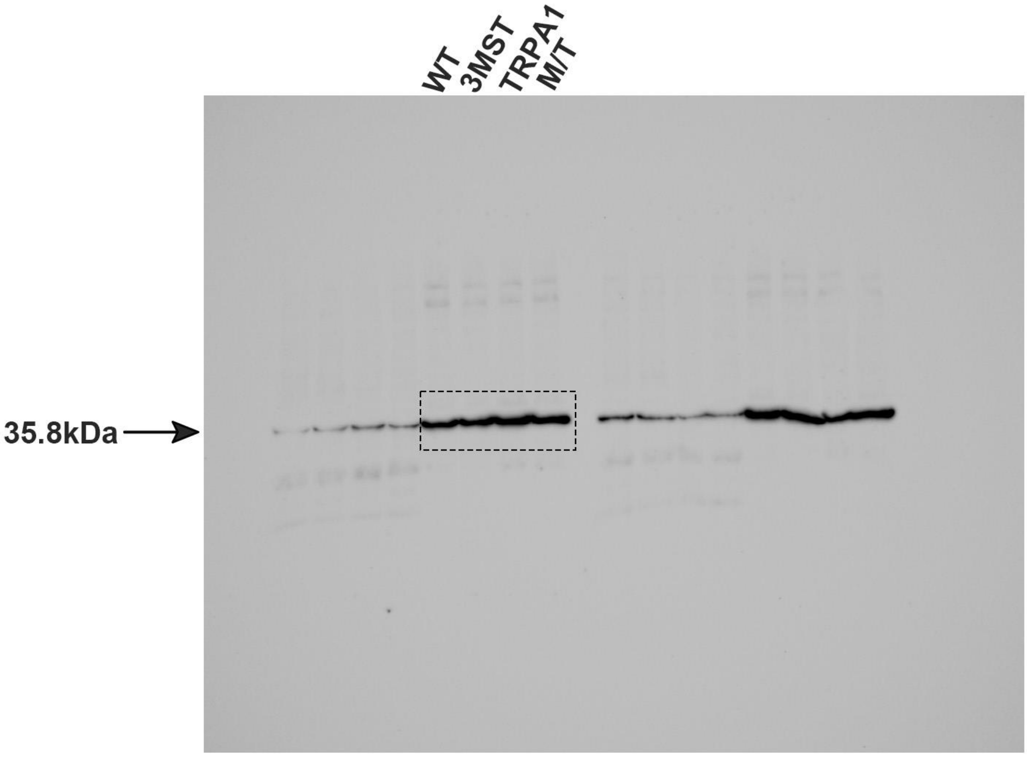

n GAPDH

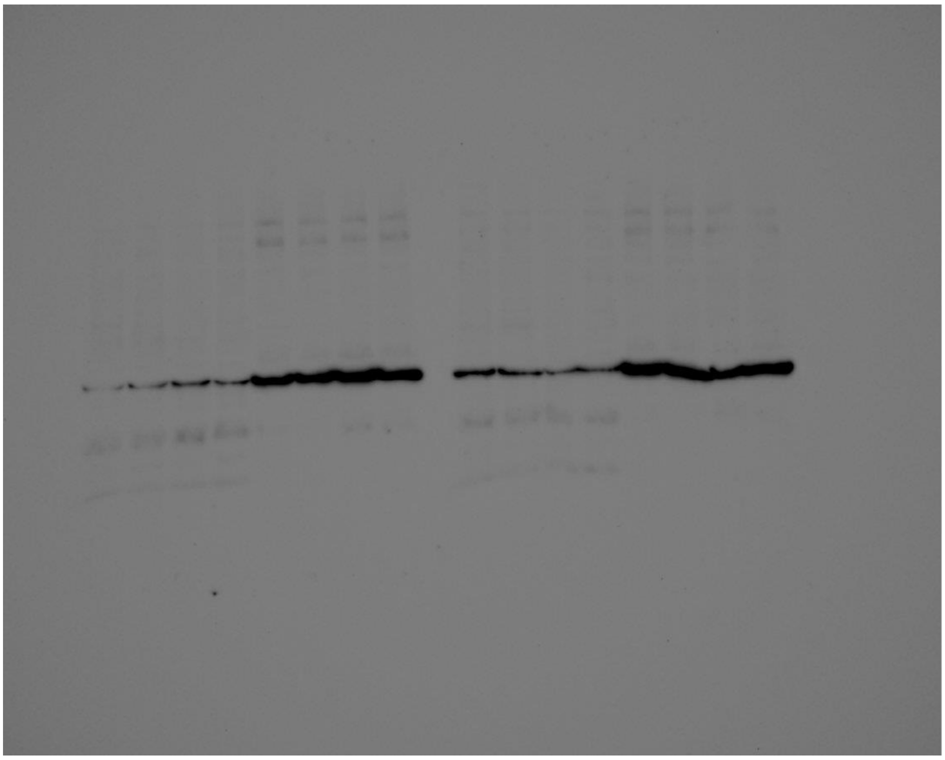

o GAPDH

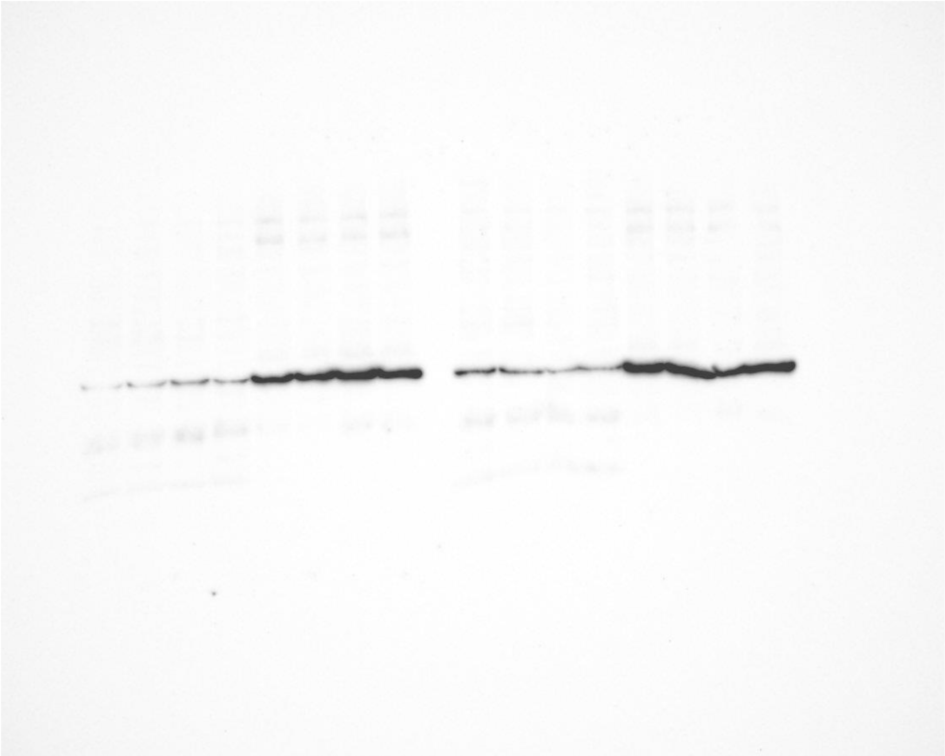

p GAPDH

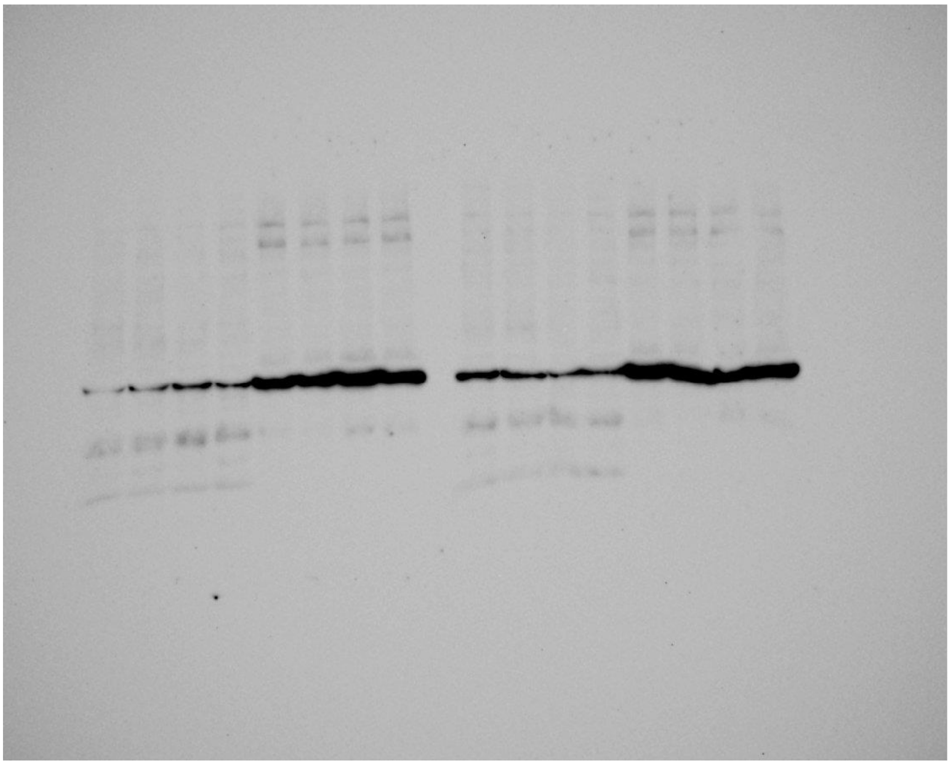

Supplementary figure 2. The images of the full length membranes of Western blot analyses of the brains shown in figure 1b and figure 1c. a-h. The blotting with antibodies against 3MST (**a**-**d**) and GAPDH (**e**-**h**). The brighter (**b**, **f**) and much brighter (**c**, **g**) images of the original blotting (**a**, **e**). The contrast was changed in **d** and **h**. The right end 4 lanes of the upper right blotting with an antibody against 3MST (**d**) and the right end 4 lanes of the left blotting with an antibody against GAPDH (**e**) were cropped and shown in Fig. 1b. **i**-**p**. The blotting with antibodies against TST (**i**-**l**) and GAPDH (**m**-**p**). The darker (**j**, **n**) and brighter (**k**, **o**) images of the original blotting (**i**, **m**). The contrast was changed in **l** and **p**. The right end 4 lanes of the left blotting with an antibody against TST (**i**) and that with an antibody against GAPDH (**m**) were cropped and shown in Fig. 1c. Although the membrane edges are not clearly visible because of the strong signals released in blotting especially with antibodies against TST and GAPDA, all images are obtained from the full length membranes of the Western blot analyses.

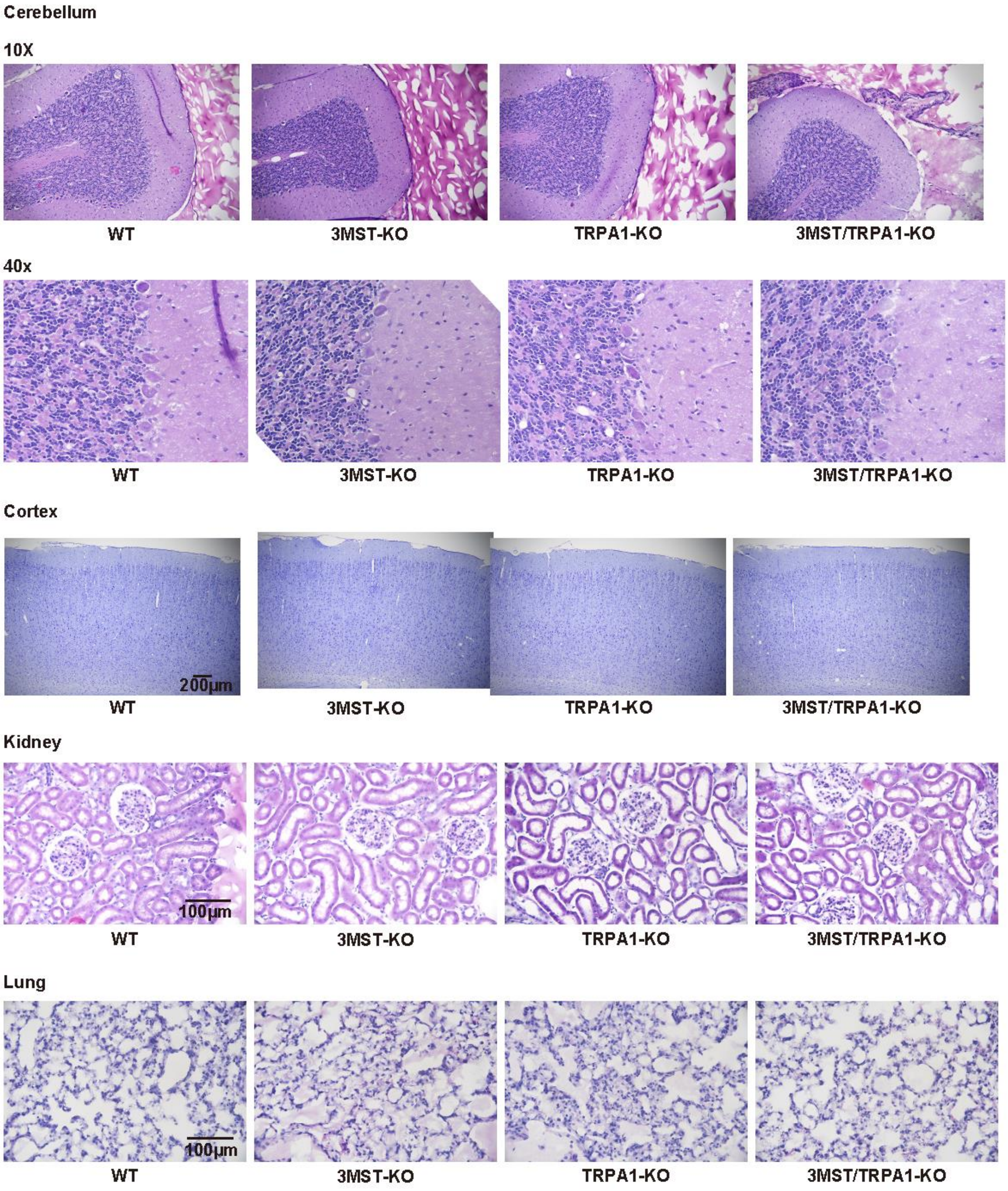

Supplementary figure 3. Morphology of the brain, kidney and lung of KO rats compared with those of the wild-type

Hemoxylin-eosin staining of cerebellum, kidney and lung, and cresyl violet staining of the cerebellum and cerebral cortex slices. These tissues were obtained from 3MST-KO, TRPA1-KO, 3MST/TRPA1 double KO, and the wild-type rats.

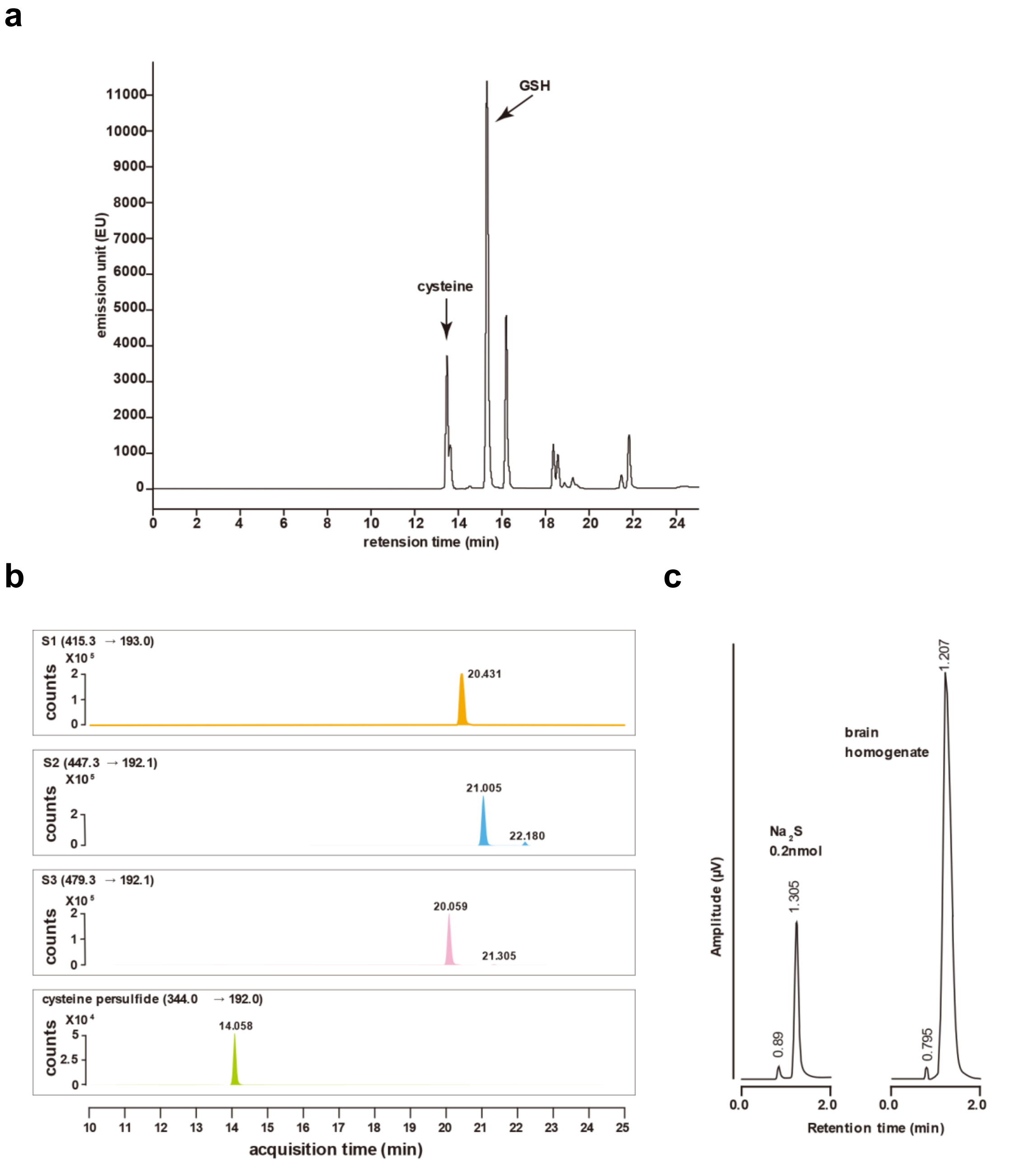

Supplementary figure 4. Chromatograms of HPLC, LC-MS/MS, and gas-chromatography. a. HPLC chromatograms of the intracellular concentrations of cysteine and glutathione (GSH) in the hippocampus. **b**. LC-MS/MS chromatograms of H_2S (S1), H_2S_2 (S2), H_2S_3 (S3), and the cysteine persulfide monobromobimane adducts. **c**. Gas chromatograms of the intracellular levels of bound (sulphane) sulfur in the hippocampus.

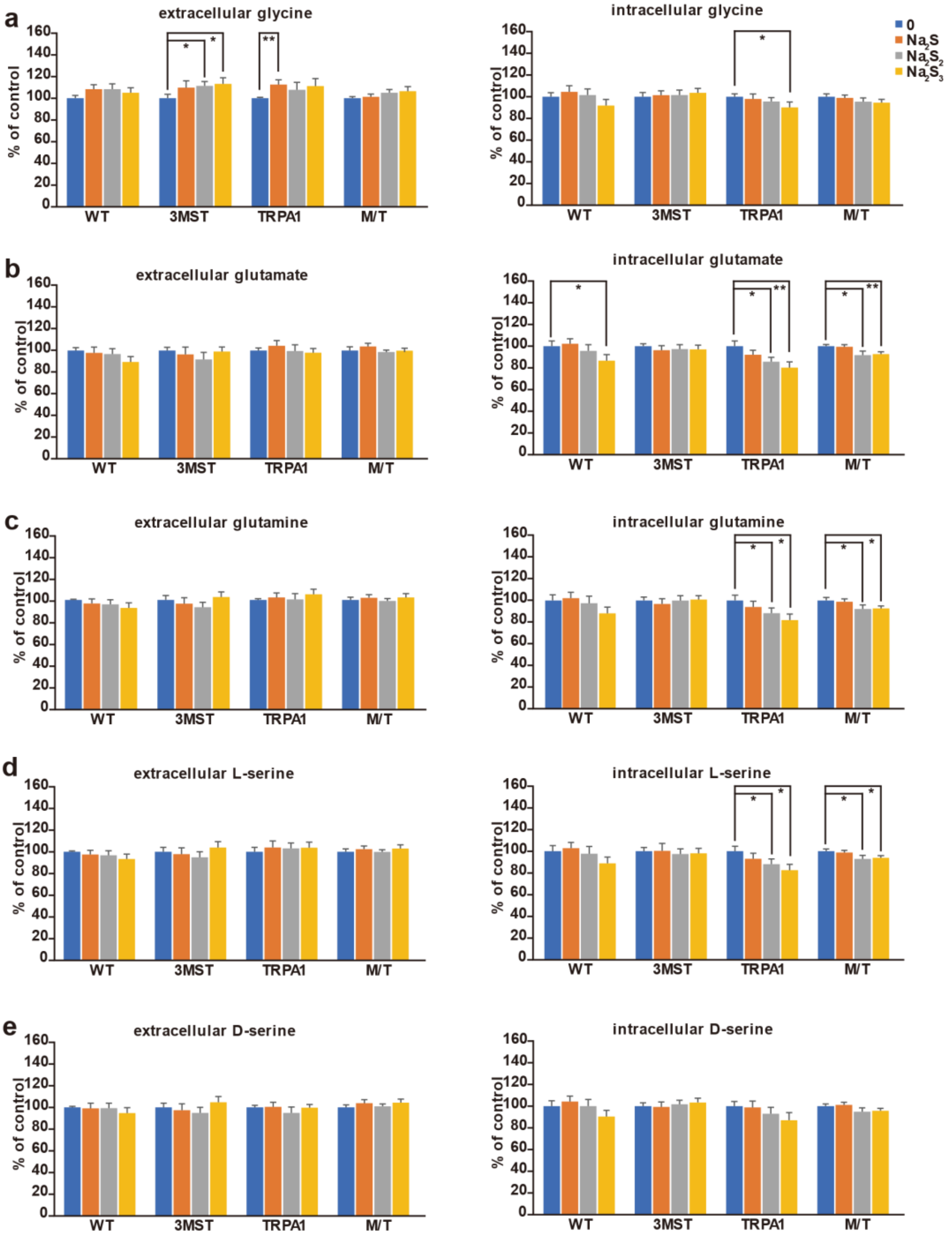

Supplementary figure 5. The release of neuro- and glio-transmitters induced by H_2S and H_2S_n from the brain cell suspension

a to **e.** The release of glycine (**a**), glutamate (**b**), glutamine (**c**), L-serine (**d**), and D-serine (**e**) from the brain cell suspension stimulated by 20 μM each of Na_2S, Na_2S_2 and Na_2S_3 and the changes in the intracellular levels of corresponding amino acid transmitters. Experiments were repeated at least 8 times. * $p < 0.05$, ** $p < 0.01$ with Student t-test. All data shown are means ± SEM.

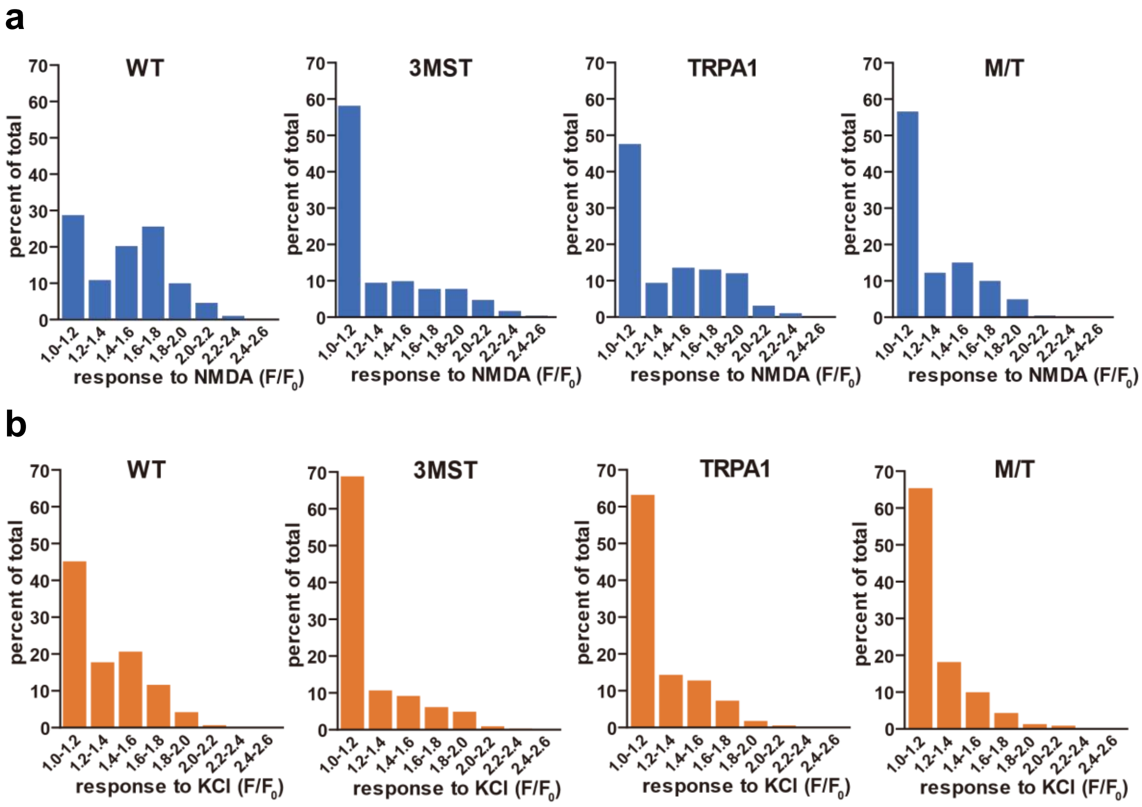

Supplementary figure 6. Sensitivity of neurons to NMDA and high K^+ prepared from 3MST-KO, TRPA1-KO, and 3MST/TRPA1-double-KO rats compared to those from the wild-type rats

a and **b.** Responses of neurons prepared from the wild-type, 3MST-KO, TRPA1-KO and 3MST/TRPA1-double-KO rats to 100 µM NMDA (**a**), and 50 mM KCl (**b**) stratified by amplitude (F/F_0).

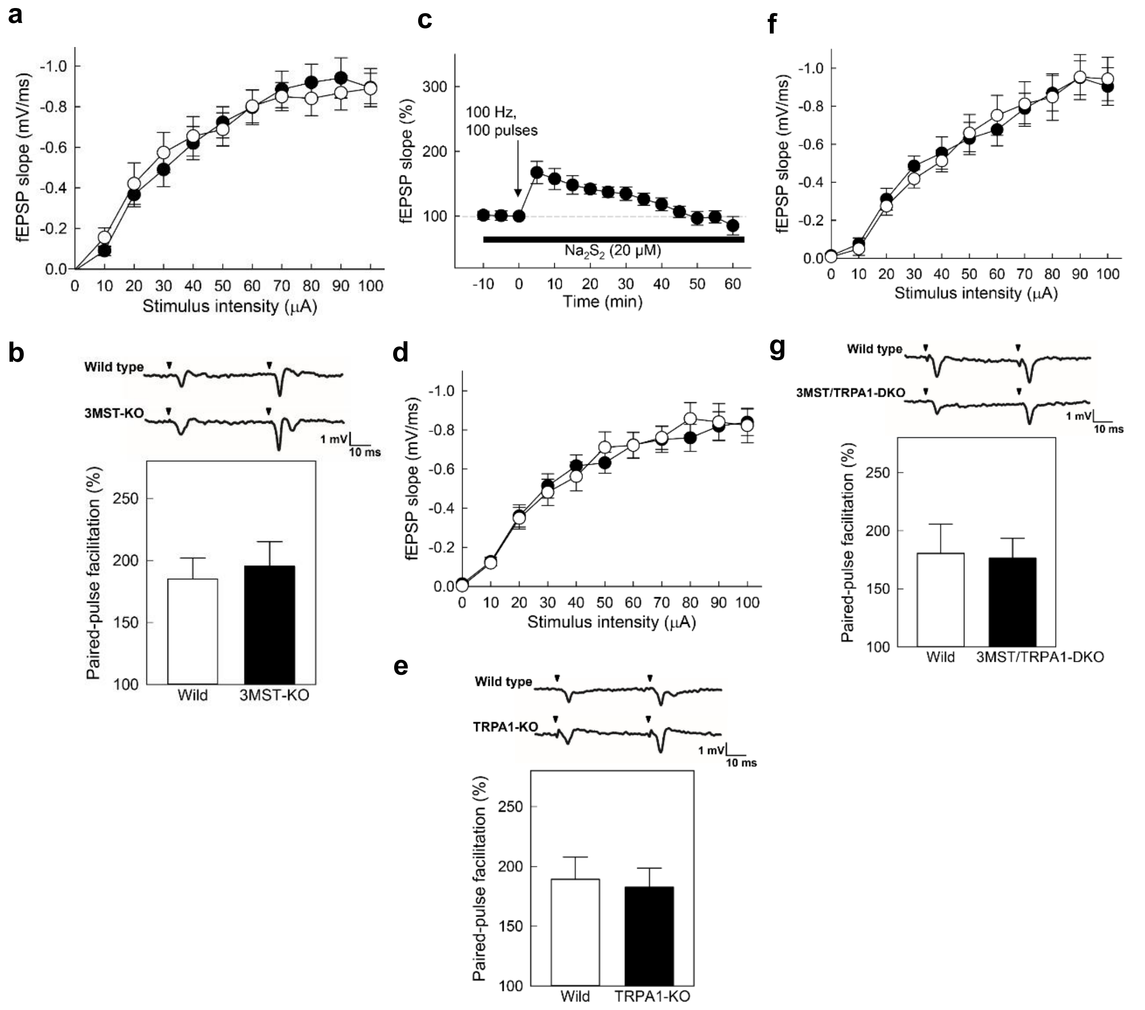

Supplementary figure 7. Induction of LTP in the hippocampus of 3MST-KO, TRPA1-KO, 3MST/TRPA1 double KO and the wild-type rats

a. Input/output relationship in 3MST-KO (closed circles, n=10) and the wild-type (open circles, n=10). **b**. Paired-pulse facilitation in 3MST-KO (n=10) and the wild-type (n=10). Paired-pulse stimulation (50 ms interval) was delivered at the arrowheads. The ratio of second fEPSP slope to the first one was indicated as pared-pulse facilitation. **c**. LTP induced in the TRPA1-KO in the presence of 20 μM Na_2S_2 (n=4). **d** and **e**. Input/output relationship (Wild: open circles, n=10; TRPA1-KO: closed circles, n=10) (**d**) and paired-pulse facilitation (Wild: n=10; TRPA1-KO: n=10) (**e**) in TRPA1-KO. **f** and **g**. Input/output relationship (Wild: open circles, n=10; 3MST/TRPA1-double KO: closed circles, n=10) (**f**) and paired-pulse facilitation (Wild: n=10; 3MST/TRPA1-double KO: n=10) (**g**) induced in 3MST/TRPA1 double KO. All data shown are means $\pm$ SEM.

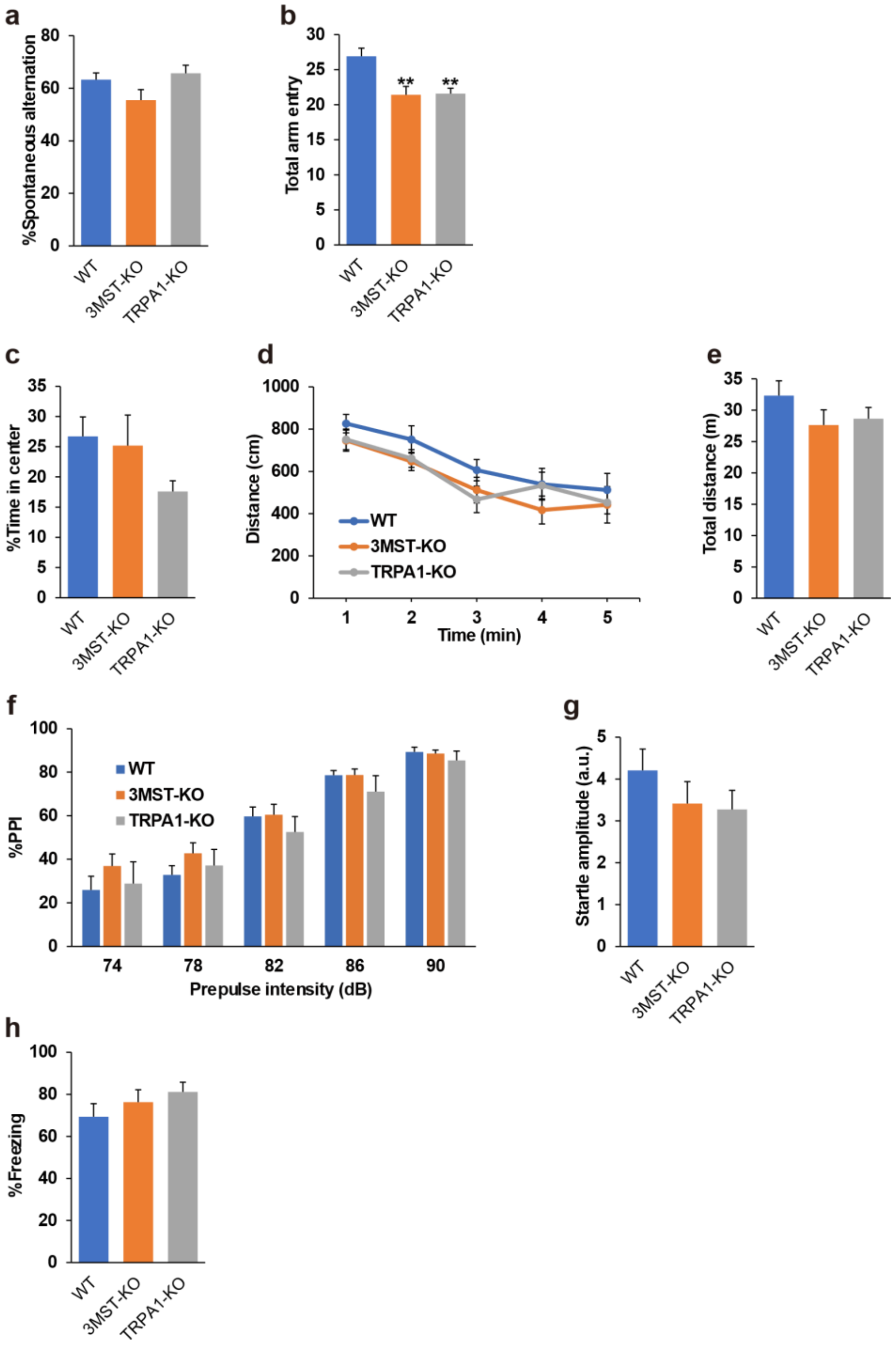

Supplementary figure 8. Behavioral tests of 3MST-KO, TRPA1-KO, and the wild-type rats a to h. Y-maze (**a** and **b**), open field (**c** to **e**), prepulse inhibition (PPI) (**f** and **g**), and contextual fear conditioning (**h**) tests performed in 3MST-KO (n =12), TRPA1-KO (n = 12), and the wild-type rats (n = 12). In Y-maze % spontaneous alternation was lower in 3MST-KO but not statistically significant (**a**), while, the total arm entry of 3MST-KO and TRPA1-KO was significantly less than that of the wild-type rats (**b**). **p < 0.01 significantly different as indicated. All data shown are means ± SEM

Supplementary table 1. ***In vitro*** **recovery (%) of Na_2S and Na_2S_2 through microdialysis probes.**

	200 μM	1 mM	10 mM
Na_2S	0.20 ± 0.09	1.08 ± 0.35	0.75 ± 0.04
Na_2S_2	0.94 ± 0.57	4.83 ± 0.83	0.84 ± 0.13

The dialysis probes (A-I-3-01; EICOM, Kyoto, Japan) used in the present study have 50,000 molecular weight cutoff with 0.3 mm outer diameter and 1 mm length (membrane exposure). It is similar to Amicon Vitafiber probe used by Kendrick [50] except for 2 mm length that has the *in vitro* recovery between 0.1 % and 7.6 % depending on molecules. All data shown are means ± SEM. The experiments were repeated three times with a new probe each.

Supplementary Methods

Y-maze

Rats were put into the center of a Y-shaped apparatus so that they faced entrance of one of the three arms and allowed to freely explore for 8 min. Total number of arm entry and percentage of spontaneous alternation were scored by an experimenter blind to rat genotypes. Spontaneous alternation was defined as entry into different arms in three consecutive choices and percent spontaneous alternation was calculated as described previously [73].

Open field

In order to investigate anxiety and baseline activity in KO rats, open field test was conducted. In the testing, rats were put into an open arena (100×100×50 cm) made of black polyvinyl chloride and allowed to explore freely for 5 min. Percentage of time spent in the center area and distance traveled of each rat was measured using SMART 3.0 video tracking software (Panlab, Barcelona, Spain).

Prepulse inhibition

In the prepulse inhibition (PPI) test, an accelerometer placed in a sound-attenuating chamber (O'Hara, Tokyo, Japan) was used to measure the startle response of rats to auditory stimuli. The test was performed as previously described [69] with minor modifications. Prior to the PPI testing, rats were placed on the accelerometer for 300 sec and habituated for white noise pulse (120 dB, 40 ms) by presenting it four times. In the PPI test, the pulses were presented alone or with prepulses of varying intensities (74/ 78/ 82/ 86/ 90 dB, 20 msec) preceding the pulse by 100 msec. The startle amplitude was expressed in arbitrary units. The test consisted of eight sessions, and six different types of trials above were conducted once per session in random order.

Contextual fear conditioning

To investigate hippocampus-dependent long-term memory, we tested rats in the contextual fear conditioning test. In the conditioning session, rats were put into a transparent conditioning chamber with stainless steel grid floor placed in a sound attenuating box (Muromachi Kikai, Tokyo, Japan). After 120 sec baseline period, electrical foot shocks (0.8 mA, 2 sec) were delivered 2 times at a 60 sec interval by foot shock generator (SGS-003, Muromachi Kikai). On the following day, memory retention test was conducted. Rats were placed in the chamber for 8 min but foot shock was not delivered. Freezing behavior during the test was measured using Time FZ software (O'Hara).

In vitro recovery of Na_2S and Na_2S_2 through the microdialysis probe

The in vitro recovery of Na_2S and Na_2S_2 through the microdialysis probes was examined. The 1.0 mm-long membrane of dialysis probes (A-I-3-01; EICOM, Kyoto, Japan) was set to submerge into 40 μl PBS (137 mM NaCl, 2.7 mM KCl, 1.76 mM KH_2PO_4 $12H_2O$, pH 7.4) in 1.0 ml Eppendorf tube and 200 μM, 1 and 10 mM Na_2S or Na_2S_2 in perfusion medium (150 mM NaCl, 2.2 mM $CaCl_2$, 4.0 mM KCl) were perfused at a flux rate of 2.0 μl/min using a syringe pump (ESP-64; EICOM). Thirty μl each of the resultant 80 μl solution was recovered to produce monobromobimane adducts. The amounts of H_2S and H_2S_2 were measured by LC-MS/MS (Agilent 6470 Triple Quad LC/MS, Santa Clara, USA). The ratio of the recovered amounts of H_2S or H_2S_2 in PBS solution to those applied in the perfusion medium was determined as the in vitro recovery (%) of Na_2S and Na_2S_2 through the probes.

Supplementary references

73. Ieraci A, Beggiato S, Ferraro L, Barbieri SS, Popoli M. Kynurenine pathway is altered in BDNF Val66Met knock-in mice: Effect of physical exercise. *Brain Behav Immun.* **89**:440-450 (2020).
